# An In Silico Target Fishing Approach to Identify Novel Ochratoxin A Hydrolyzing Enzyme

**DOI:** 10.3390/toxins12040258

**Published:** 2020-04-16

**Authors:** Luca Dellafiora, Christoph Gonaus, Barbara Streit, Gianni Galaverna, Wulf-Dieter Moll, Gudrun Vogtentanz, Gerd Schatzmayr, Chiara Dall’Asta, Shreenath Prasad

**Affiliations:** 1 Department of Food and Drug, University of Parma, Parco Area delle Scienze 27/A, 43124 Parma, Italy; gianni.galaverna@unipr.it (G.G.); chiara.dallasta@unipr.it (C.D.); 2 BIOMIN Research Centre, Technopark 1, 3430 Tulln an der Donau, Austria; christoph.gonaus@biomin.net (C.G.); barbara.streit@biomin.net (B.S.); dieter.moll@biomin.net (W.-D.M.); gudrun.vogtentanz@biomin.net (G.V.); gerd.schatzmayr@biomin.net (G.S.)

**Keywords:** ochratoxin A, ochratoxin alpha, in silico screening, enzymatic detoxification, mitigation, mycotoxin

## Abstract

Ochratoxin A (OTA), a mycotoxin that is of utmost concern in food and feed safety, is produced by fungal species that mainly belong to the *Aspergillus* and *Penicillium* genera. The development of mitigation strategies to reduce OTA content along the supply chains is key to ensuring safer production of food and feed. Enzyme-based strategies are among the most promising methods due to their specificity, efficacy, and multi-situ applicability. In particular, some enzymes are already known for hydrolyzing OTA into ochratoxin alpha (OTα) and phenylalanine (Phe), eventually resulting in detoxification action. Therefore, the discovery of novel OTA hydrolyzing enzymes, along with the advancement of an innovative approach for their identification, could provide a broader basis to develop more effective mitigating strategies in the future. In the present study, a hybrid *in silico/in vitro* workflow coupling virtual screening with enzymatic assays was applied in order to identify novel OTA hydrolyzing enzymes. Among the various hits, porcine carboxypeptidase B was identified for the first time as an effective OTA hydrolyzing enzyme. The successful experimental endorsement of findings of the workflow confirms that the presented strategy is suitable for identifying novel OTA hydrolyzing enzymes, and it might be relevant for the discovery of other mycotoxin- mitigating enzymes.

## 1. Introduction

**Figure 1:**
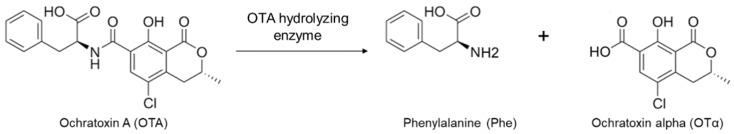
Scheme of Ochratoxin A hydrolysis into phenylalanine and Ochratoxin α.

Ochratoxin A (OTA) is a low molecular-weight secondary metabolite that is produced by fungal species mainly belonging to *Aspergillus* and *Penicillium* genera [1,2]. From a chemical point of view, OTA is a phenylalanyl derivative of a substituted isocoumarin, referred to as (R)-N-[(5-Chloro-3,4-dihydro-8-hydroxy-3-methyl-1-oxo-1H-2-benzopyran-7-yl)-carbonyl]-L-phenylalanine (Figure 1). OTA is the most toxic member of the ochratoxins group [3] and it is among the mycotoxins that are of biggest concern for food and feed safety due to many adverse effects on humans and animals [4,5]. For example, nephrotoxicity, immunotoxicity, and carcinogenicity have been described among the possible elicited effects. In this respect, OTA has been classified by the International Agency of Research on Cancer (IARC) in Group 2B as a possible human carcinogen [6].

OTA might broadly contaminate different types of food and feed, including cereals, cereal-based products, wine, vinegar, fruits, beans, dried food products, spices, etc., as OTA-producing fungi may grow at pre- and post-harvest stages of several crops. In addition, OTA can be found also in egg, meat, and dairy products due to carry over phenomena when the animals are fed with contaminated feed [7,8,9]. The European Commission set limits in several food and feed products in the range of 0.5–10 μg/kg (EC, 1881/2006) [10] and 10–250 μg/kg (EC, 2016/1319) [11], respectively, due to the risk to health and well-being of humans and animals associated with OTA.

In general, the application of mitigation strategies to further reduce the content of mycotoxins in compliant food and feed batches is receiving increasing interest to ensure a safer supply chain [12]. Specifically, mitigation strategies refer to processes applied along the production chain that may reduce the abundance of specific mycotoxins via chemical, physical, or biological means, with the latter including bacteria, yeasts, molds, and enzymes [13,14,15]. Enzyme-based strategies merit attention thanks to their efficacy in detoxifying specific mycotoxins, including OTA, aflatoxin B1, fumonisin B_1_, and zearalenone, and their theoretical multi-situ applicability along the production chain [15,16,17,18]. In addition, they have been shown to reduce the toxic load of certain mycotoxins *in vivo* when administered to livestock [19]. Nonetheless, the identification of effective enzymes can be labor intensive and time consuming; therefore, a straightforward framework of analysis is required to support more efficient enzyme identification strategies.

For the purpose of OTA mitigation, enzymatic strategies are of particular interest due to the thermal stability of OTA that might reduce the degrading efficacy of thermal treatments [1]. Especially, it has already been demonstrated that OTA is prone to undergo hydrolysis by various hydrolases, including peptidase enzymes [20,21,22,23], releasing phenylalanine (Phe), and the less toxic derivative ochratoxin α (OTα) [24] as reaction products (Figure 1).

**Figure 2:**
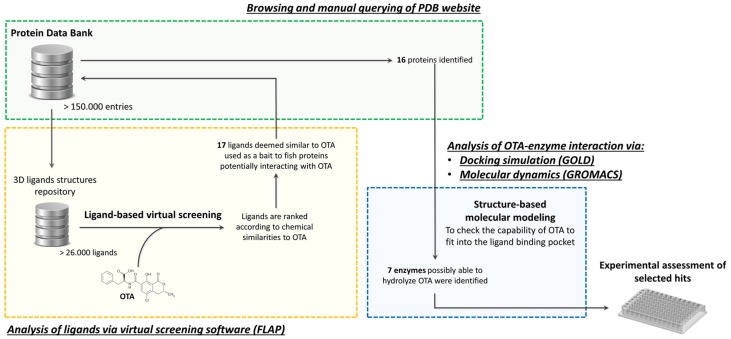
Schematic representation of the workflow used in the *in silico* target fishing study to identify Ochratoxin A (OTA) hydrolyzing enzyme.

In the present work, we applied a previously defined *in silico* target fishing approach (Figure 2), which already succeeded in identifying proteins that are able to interact with small molecules [25,26], in order to identify novel enzymes for OTA degradation (Figure 1).

The *in silico* target fishing approach that was used in this study consists of a computer-driven high-throughput screening of wide libraries of Protein Data Bank (RCSB PDB; https://www.rcsb.org) entries to identify proteins that are possibly able to either interact with or transform OTA (Figure 2). The underlying basic principle of this approach relies on the capability of chemically similar molecules to interact with the same targets [27]. In more detail, the repository of ligands in the bound state with the proteins/enzymes being recorded in the PDB was virtually screened to find those that were most similar to OTA. The chemical similarity between OTA and ligands from PDB could be theoretically used as a “bait” in the fishing process in order to identify proteins/enzymes that are also potentially able to interact with OTA. A structure-based molecular modeling approach was then used to thoroughly assess each OTA-enzyme complex formation according to a procedure that was defined by Dellafiora and coworkers [25]. In the present work, we discuss the outcome *of in silico* target fishing (Figure 2), as well as the enzymatic activity of identified potential enzyme targets for hydrolysis of OTA into OTα and Phe (Figure 1).

## 2. Results and Discussion

### 2.1. Ligand Dabase Anatomy

The final ligand-database grouped 26.036 out of the 26.868 ligands that were downloaded from PDB (Figure 2), as all the metal complexes (including those with transition metals) and/or molecules with non-canonical binding geometries were automatically discarded by the software. The eventuality of missing proteins possibly able to transform/bind OTA was considered not likely due to stark chemical differences between the discarded molecules and OTA.

**Figure 3:**
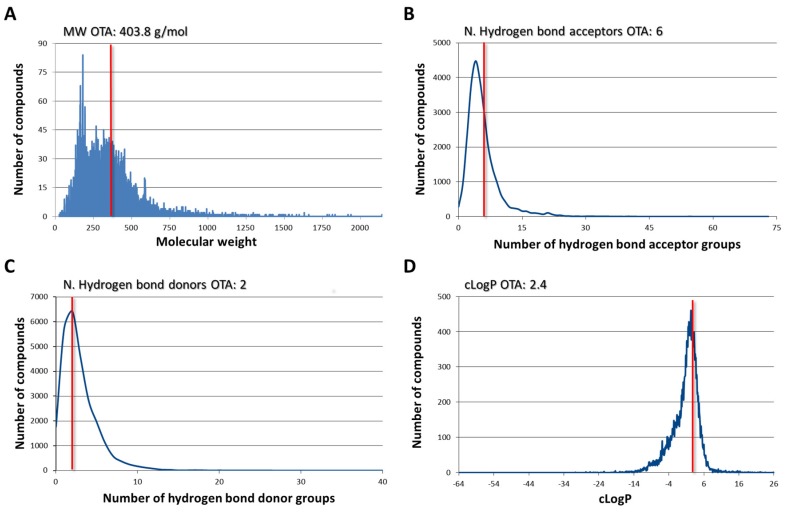
Description of chemical space represented in the ligands database in terms of (**A**) molecular weight (MW), (**B**) number (N) of hydrogen bond acceptor groups, (**C**) number (N) of hydrogen bond donor groups, and (**D**) calculated LogP (cLogP). Red lines highlight the position of Ochratoxin A (OTA).

The description of chemical space represented in the final database of 26.036 ligands in terms of molecular weight (MW), the number of hydrogen bond donors/acceptors, and calculated LogP (cLogP) is reported in Figure 3. OTA has MW of 403.8 g/mol, two and six hydrogen bond donors and acceptors, respectively, and a cLogP of 2.4. OTA features are well represented, which indicates the appropriate coverage of database to discover ligands that are similar to OTA, as shown in Figure 3.

### 2.2. Ligand-Based Virtual Screening (VS)

The FLAP software screened 26.011 ligands out of 26.036 ligands; 25 ligands were excluded from the analysis due to processing errors. The selection of compounds most similar to OTA and, consequently, the identification of respective protein (including enzyme) targets possibly able to interact/transform OTA relied on the consensus scoring of five different VSs (see Section 4.2 for further details; Figure 2). For each VS, ligands were sorted according to “distance”, “Global Sum”, and “Global Product” parameter (see Section 4.2 for further details). Subsequently, all of the ligands within percentage score variation from the best scored ligand up to 22%, 70%, and 26% for “distance”, “Global Sum”, or “Global Product”, respectively, were inspected. Percentage thresholds were arbitrarily set to only focus on the most similar compounds to achieve a manageable number of entries for the following steps. Consequently, 86 non-redundant ligands and their 145 proteins were identified (Table S1, Supporting Material) as possible candidates that are able to interact/transform OTA. Nevertheless, the subsequent manual inspection of ligands and the subsequent selection of proteins consisted of: i) an expert visual analysis of ligands structure, which led to exclude structures reasonably unrelated to OTA from further analysis; and, ii) the evaluation of the proteins type considering their relevance to the purpose of this work to identify novel OTA hydrolyzing enzyme. In particular, the inclusion criteria of proteins relied either on structural analogies or on the existence of reasonably conserved mechanisms of catalysis with respect to already characterized OTA hydrolyzing enzymes, and potential non-OTA hydrolyzing enzymes as well as potential OTA binders (Table 1). As an example, transferases, ligases, phosphatases, DNA/RNAes, transcriptional factors, cytochromes, proteins with unknown function, and unspecific enzymes that are involved in sugars and lipids metabolism, which could not be directly related to OTA transformation/interaction, were discarded from being processed further. Proteins from either animal or human pathogens or already known for poisonous activity were also excluded. This expert filtering identified 17 different ligands and 16 non-redundant proteins (Table 1). The proteins list (Table 1) included 12 hydrolases (potential OTA hydrolyzing enzymes), two proteins (potential OTA binders), and two non-OTA hydrolyzing enzymes (perhaps responsible for catalyzing new OTA transformation reaction).

It is worth noticing that proteins/enzymes already known to bind/hydrolyze OTA were included in the list of entries identified (Table 1). In particular, bovine carboxypeptidase A (CPA) and thermolysin were already shown to hydrolyze OTA into OTα and phenylalanine [20,21,28], while serum albumin has been repeatedly described as OTA binder [29,30,31]. These results corroborate the efficacy of the *in silico* approach presented here to identify proteins that are able to interact/transform OTA.

When considering the aim of the present work to identify novel OTA hydrolyzing enzymes, the already known OTA hydrolyzing enzymes (thermolysin & CPA), known OTA binding protein (serum albumin), potential new OTA binding protein (Transthyretin), and potential non-hydrolyzing OTA transforming enzymes (Salicylate 1,2-dioxygenase and N-acyl amino racemase) (Table 1) were excluded from the next step—structure-based molecular modeling. The remaining ten potential OTA hydrolyzing enzymes (Table 1; CPT, CPB, Ser-CP II, neprilysin, urokinase, endothiapepsin, cathepsin A, MMP-12, BACE 1, and pancreatic elastase) were further evaluated for their OTA hydrolyzing potential by structure-based molecular modeling.

### 2.3. Structure-Based Molecular Modeling

Structure-based molecular modeling of selected ten potential OTA hydrolyzing enzymes relied on two steps: 1) docking studies, to achieve a plausible binding pose of OTA within each protein, followed by 2) molecular dynamic (MD) simulations (20 nanoseconds), in order to check the capability of OTA to persist within the respective catalytic/binding site (Figure 2). Docking analysis provided reasonable arrangements of OTA within each binding site. Therefore, the collected scores were not used in a comparative way to estimate the binding capability of ligands neither quantitatively, nor qualitatively (scores are summarized in Table S2; Supporting Material). With respect to MD simulations, the geometrical stability of protein-OTA complexes was assessed measuring the Root Mean Square Deviation (RMSD) and checking the OTA trajectories, as previously reported [32] (further details are reported in Supporting Material). The capability of persisting over the time within enzymes in a conformation likely prone to undergo the enzymatic reaction was considered to be the rationale to hypothesize a substrate-like behavior of OTA.

Before running MD simulations, OTA poses calculated in docking studies were compared to those of the already characterized substrates to preliminarily assess the likeliness of OTA interaction as a substrate. In addition, any protein-OTA complex that was unlikely to hydrolyze OTA did not undergo validation through MD analysis. In this respect, pancreatic elastase was excluded from being processed with MD simulations, because the mode of OTA binding that was calculated by docking simulations was considered as not likely to undergo hydrolysis. Specifically, the coumarin-like moiety of OTA exceeded the space available for ligand, moving OTA away from the catalytic site and forcing it to adopt an unlikely orientation to undergo the reaction. Conversely, the remaining nine proteins (i.e., CPT, CPB, Ser-CP II, neprilysin, urokinase, endothiapepsin, cathepsin A, MMP-12, BACE 1) that are listed in Table 1 that were never tested before for their capability to hydrolyze OTA were considered for MD simulations, as detailed below.

#### 2.3.1. Carboxypeptidase T (CPT)

CPT is a bacterial carboxypeptidase, which is homologous to the bovine carboxypeptidase A (CPA), that is among the first enzymes identified for OTA-hydrolyzing activity [20,21]. In spite of the relatively low primary protein sequence identity of 26.6% (is hereinafter referred to as % sequence identity), CPT and CPA are both able to bind the same competitive inhibitor CXA (phenylalanine-N-sulfonate) (Table 1), suggesting the possibility to have some ligands in common. Docking studies showed the capability of OTA to adopt a conformation that is likely prone to undergo hydrolysis of the amide bond (as per Figure 1), which was well oriented with respect to the catalytic core (95.7 GOLDScore units), and resembling the theoretical orientation within CPA (Figure S1, Supporting Material). MD analysis described a good stability of both the enzyme and OTA throughout the simulation. In addition, the analysis of trajectories revealed that OTA stably persisted within the catalytic site (Figure S2, Supporting Material). These points could suggest a functional conservation of CPT as OTA hydrolyzing enzyme.

#### 2.3.2. Carboxypeptidase B (CPB)

CPB is a porcine homolog to the known OTA-hydrolyzing enzyme CPA [20,21], and it shares 44.7% sequence identity. CPB is reported to also bind the competitive inhibitor of CPA, as described above for CPT (CXA, phenylalanine-N-sulfonate; Table 1), also suggesting, in this case, the possibility to have some ligands in common. According to docking studies, OTA was able to interact well with the catalytic site (95.3 GOLDScore units; Figure S3B, Supporting Material), similarly to the pose observed within CPT (Figure S1C, Supporting Material). MD analysis described a good geometrical stability of the enzyme, while the geometry of OTA interaction was found to be less stable (Figure S4A and Figure S4B, Supporting Material). Nevertheless, the analysis of OTA trajectories (i.e., the path of OTA molecule movement within the binding pocket of enzyme) revealed that the coumarin-like moiety was mainly reorganized during the simulation, while both the amidic bond and the Phe moiety were kept stable and theoretically well organized with respect to the catalytic site to undergo the reaction (Figure S4C, Supporting Material). Notably, the persistence of OTA amidic bond close to the catalytic Zn ion, in spite of the relatively high mobility of the rest of the molecule, served as geometrical rationale for hypothesizing a substrate-like behavior of OTA. On this basis, CPB might have a functional OTA hydrolyzing activity.

#### 2.3.3. Serine Carboxypeptidase II (Ser-CP II)

This enzyme shows structural analogies with the OTA-hydrolyzing enzyme carboxypeptidase Y (CPY) from baker’s yeast [21], and it shares 28.9% sequence identity. Specifically, CPY could not be identified in the ligand-based virtual screening, as the CPY structures available in the PDB at the time of investigation were not bound to any ligand (i.e., the unbound state). OTA recorded a plausible orientation to undergo hydrolysis (54.7 GOLDScore units), although with a slightly different orientation in respect to that expected within CPY, according to docking results (Figure S5, Supporting Material). MD analysis revealed a good stability of both OTA and Ser-CP II; however, a clear outward trajectory of OTA from the catalytic site was observed (Figure S6, Supporting Material). On this basis, the interaction of OTA with Ser-CP II was considered not sufficiently stable and its substrate-like behavior was considered to be not likely. When considering these points, Ser-CP II might not be an efficient potential OTA hydrolyzing enzyme.

#### 2.3.4. Neprilysin

This enzyme is a zinc-dependent endopeptidase that is able to hydrolyze peptides up to 50 residues, and it does not share homology with any known OTA hydrolyzing enzymes so far. OTA (87.9 GOLDScore units) showed a good orientation to undergo reaction as the amidic bond was placed close to the catalytic zinc ion, according to docking results (Figure S7, Supporting Material). MD analysis revealed that the protein geometry was stable throughout the simulation while the geometry of OTA interaction was found to be less stable (Figure S8A,B, Supporting Material). In particular, the analysis of trajectories revealed that OTA had an early increase of RMSD mainly due to the reorganization of the coumarin-like moiety, but the overall organization of the amidic bond with respect to the Zn ion was kept stable throughout the simulation (Figure S8C, Supporting Material). On this basis, this enzyme could be considered as a potential OTA hydrolyzing enzyme.

#### 2.3.5. Urokinase

This enzyme is a serine protease that is related to the already characterized OTA-hydrolyzing enzyme bovine alpha chymotrypsin [20,33], and it shares 30% sequence identity. Notably, bovine alpha chymotrypsin was not identified during the ligand-based screening, although it was annotated in PDB in a ligand-bound state, due to the marked chemical difference between the ligands bound and OTA. This result highlighted that the possibility to screen out effective enzymes depends on the accessibility of structural information for the enzymes to be screened (i.e., availability of a wide array of protein structures in PDB) and by the number and chemical heterogeneity of molecules that were bound to a given enzyme. When considering the fast and continuous growth of PDB database in terms of new and heterogeneous records of proteins and ligands, this inherent bias will be progressively overcome, which makes this kind of approach increasingly effective in the future. According to docking studies, OTA (64.3 GOLDScore units) posed the amidic bond in a reasonably well oriented position in the binding pocket of urokinase to undergo hydrolysis (Figure S9, Supporting Material). MD analysis revealed a good stability of the enzyme, while the geometry of OTA interaction was found to be less stable over time (Figure S10, Supporting Material). The analysis of OTA trajectories showed that the Phe moiety was the most mobile region, while the remaining part of the molecule, including the amidic bond, was kept stable during the considered timeframe. The persistence of OTA amidic bond close to the catalytic core of urokinase was used as a geometrical rationale to hypothesize a substrate-like behavior of OTA, as shown for the OTA-CPB complex. Moreover, OTA was found to be stably buried within the catalytic site during the all simulation. On this basis, this enzyme might have functional OTA hydrolyzing activity.

#### 2.3.6. Endothiapepsin

This enzyme is a pepsin-like aspartic protease. According to docking studies, OTA scored 62.4 GOLDScore units and it arranged the amidic bond close to the catalytic core, possibly pointing to an organization that is prone to undergoing hydrolysis (Figure S11, Supporting Material). However, MD analysis revealed a marked geometrical instability of both OTA and protein, while the trajectory analysis described a clear outward movement of OTA from the binding site (Figure S12, Supporting Material). On this basis, the interaction of OTA with protein was considered to be unstable and its substrate-like behavior was considered as not likely. Therefore, this enzyme could not be considered as a potential OTA hydrolyzing enzyme.

#### 2.3.7. Cathepsin A

This enzyme is a broad-spectrum serine protease homologous to the already characterized OTA-hydrolyzing enzyme carboxypeptidase Y (CPY) [21], and it shares 29% sequence identity. According to docking studies, OTA scored 59.6 GOLDScore units and adopted a binding pose resembling the one that was observed within the Ser-CP II (see above) arranging the amidic bond in an orientation possibly suitable to undergoing hydrolysis (Figure S13, Supporting Material). MD analysis revealed that both the protein and OTA showed a slight increase of mobility along the considered timeframe. In addition, the analysis of OTA trajectory did not show a geometrically stable interaction with the binding/catalytic site (Figure S14, Supporting Material). Nonetheless, the instability of the Cathepsin A-OTA complex could have been due to the incompleteness of the model used. Specifically, the conversion of cathepsin A from precursor to the mature enzyme requires a proteolytic cleavage that releases two chains of 32 and 20 kDa [34]. The N-terminus of the light chain is proximal to the catalytic site, and all of the structures available in PDB had portions unresolved in that region (PDB accessed on December 17, 2018). In particular, the seven N-terminal residues were missing in the structure used here (PDB code 4AZ0). In addition, there are no structural clues available so far to drive a reasonably reliable knowledge-based modeling of the missing part. Therefore, the calculation was run while using the incomplete model, rather than modeling the missing region, in order to avoid the introduction of structural biases and model artifacts. Based on these points, cathepsin A might have OTA hydrolyzing activity despite the computational outcome collected.

#### 2.3.8. Matrix Metalloproteinase 12 (MMP-12)

MMP-12 is a Zn- and Ca-dependent protease that cleaves a broad range of substrates. Like neprilysin, MMP-12 also does not share homology with any known OTA hydrolyzing enzymes. OTA scored 59.7 GOLDScore units and it arranged the amidic bond with respect to the catalytic metal in a possibly suitable orientation to undergo hydrolysis, according to docking studies (Figure S15, Supporting Material). MD analysis revealed that the complex was overall stable, and the analysis of OTA trajectory showed that it stably persisted close to the catalytic site properly arranging the amidic bond to the catalytic metal to undergo hydrolysis (Figure S16, Supporting Material). On this basis, this enzyme might have potential OTA hydrolyzing activity.

#### 2.3.9. Beta-Secretase 1 (BACE1)

BACE1 is an endopeptidase with a relatively non-stringent specificity that is homologous to the aspartic proteases of the pepsin family. According to docking studies, OTA scored 58.2 GOLDScore units and it arranged the amidic bond toward the catalytic site with an orientation possibly compatible to undergo hydrolysis (Figure S17, Supporting Material). MD analysis revealed that the complex was stabilized toward the end of simulation, while the analysis of trajectories described an outward movement followed by an inward migration of OTA toward the binding site in the last part of the simulation (Figure S18, Supporting Material). Taking the observed capability of OTA to re-enter the catalytic site into account, BACE1 might have functional OTA hydrolyzing activity.

### 2.4. Confirmation of OTA Hydrolyzing Activity of Selected Enzymes

Among the seven potential OTA hydrolyzing enzymes that were identified by *in silico* studies (see above), five were commercially available (i.e., neprilysin, MMP12, CPB, urokinase, and BACE-1), and they were selected for the experimental assessment of their OTA hydrolyzing activity (Figure 2). The OTA hydrolyzing activity was measured checking the disappearance of OTA and the appearance of OTα in the reaction mix after 180 minutes of incubation in comparison to reaction controls, where each enzyme was omitted from the reaction mixture (further details are reported in material and methods, Section 4.4, Figure 1). The appearance of OTα peak was detected in the reaction mix of CPB only, thus confirming its OTA hydrolyzing activity. Notably, the initial amount of OTA was decreased to 93.1 ng/mL after 180 minutes of incubation in comparison to the estimated concentration 97.0 ng/mL of OTA negative reaction control, while OTα appeared around 2.79 ng/mL (Figure S19, Supporting Material). In contrast, the positive control, CPA, resulted in >95% transformation of OTA into OTα. When considering the enzyme concentration, reaction time, percentage transformation of OTA into OTα, etc., it seems that CPB is approximately eightfold less active in comparison to the CPA. However, in comparison to a previous report on CPA (where almost 100% transformation of 1000 ng/mL OTA into OTα by 0.5mg/ml CPA in 40 min. at 37 °C is reported [21]), CPB is approximately 90-fold less active. Further, it is worth expressing recombinant CPB for the comprehensive biochemical characterization.

This is the first report on the OTA hydrolyzing activity of pig CPB, which seems to be comparably in line with the one reported in the literature for CPA [23,33]. In contrast, the OTA hydrolyzed product OTα was not detected in the reaction mixture of the other four tested enzymes (neprilysin, MMP-12, urokinase, and BACE-1), which indicates that either these enzymes have no OTA hydrolyzing activity at all, or that an appreciable hydrolysis was not possible under these experimental conditions. Additionally, a very low-efficiency release of OTα below the limit of detection cannot be excluded. Therefore, testing these enzymes with a different set of experimental conditions would be warranted. Moreover, keeping in mind that all of these enzymes were identified using bait molecules with inhibitory activity, their possible inhibition by OTA would also need to be assessed. CPT and cathepsin A, which both gave promising results in the computational assessment, were not commercially available. It would be interesting to produce them by recombinant expression and test their OTA hydrolyzing activity.

**Figure 4:**
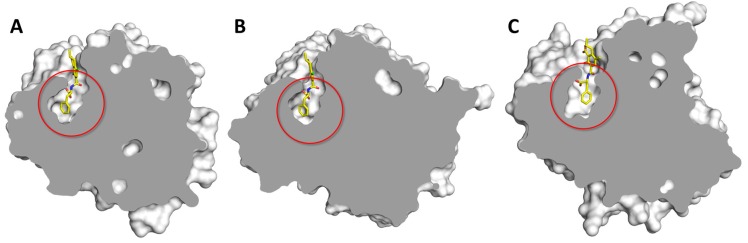
Graphical representation of OTA in complex with carboxypeptidase B (CPB) (**A**), carboxypeptidase A (CPA) (**B**), and carboxypeptidase T (CPT) (**C**). Proteins are represented in gray cut surface while OTA is represented in yellow sticks. The red ring indicates the position of the buried cleft receiving the Phe moiety of OTA.

The close inspection of the computed binding poses of OTA within the experimentally tested enzymes revealed a substantial difference between the arrangement of the hydrolytically active CPB and the other four non-active enzymes. Specifically, only the interaction with CPB was found to be spatially constrained with respect to the catalytic site with the Phe moiety (including the carboxylic group) buried within a surface cleft of the enzyme with a particularly good shape fitting (Figure 4A). This architecture was also found for the computed OTA-CPA complex (Figure 4B), suggesting a buried anchoring of Phe as an important feature leading to OTA hydrolysis by peptidases. Based on this, a sterical restrain further limiting the movements of OTA within the catalytic site is likely to foster its hydrolysis, despite the fact that MD simulations did not reveal significant detachments from the catalytic sites of any of the enzymes selected for experimental trials. Concerning the possibility to check this specific point via MDs, either an extension of the timeframe calculated (i.e., > 20 nanoseconds) or the application of a different MD approach (e.g., steered MD simulation) can be considered in future studies to better distinguish non-effective enzymes monitoring OTA detachment from the catalytic site. However, on the basis of the evidence collected here, the sterical restraint mentioned above might provide a convincing rational to explain the activity of CPB and the inactivity of the other four enzymes tested (i.e., neprilysin, urokinase, BACE-1, and MMP12). Concerning the other two enzymes that were identified by *in silico* screening but not tested experimentally (i.e., CPT and cathepsin A), the binding mode of OTA within CPT starkly resembled the one within CPA and CPB (Figure 4C), further pointing to likeliness of CPT as OTA hydrolyzing enzyme.

## 3. Conclusions

OTA might pose a severe risk to health of humans and animals. Therefore, the mitigation of OTA content in food and feed is of utmost importance to ensure an increasingly safe supply chain. In this context, the use of enzyme-based mitigation strategies is considered to be among the most promising approaches to minimize the content of OTA in the final products. On the other hand, the identification of suitable enzymes can be challenging and the development of a straightforward framework of analysis to support the discovery of novel effective enzymes is increasingly desirable.

In this work, we applied a hybrid *in silico/in vitro* approach to identify novel enzymes that are able to degrade OTA upon hydrolysis into OTα and Phe (Figure 1 and Figure 2). A virtual database of more than 150.000 proteins from the Protein Data Bank was screened while using a combined approach based on ligand-based virtual screening and structure-based molecular modeling. Most of the proteins already characterized as being able to either hydrolyze or bind OTA were identified in the early stages of the workflow presented here, confirming the reliability of the proposed approach. Additionally, seven potential OTA hydrolyzing enzymes never considered before for OTA hydrolyzing activity were identified by the *in silico* target fishing study. Among them, five peptidases were experimentally screened for OTA hydrolyzing activity, which was confirmed for the porcine carboxypeptidase B (CPB) for the first time. This enzyme showed an activity fairly resembling the one of CPA (one of the OTA hydrolyzing enzymes previously characterized), and it could be considered as a rational evidence-based starting point for future engineering strategies to improve its hydrolyzing activity.

Taken together, the presented results provide a reliable knowledge-based foundation for developing further enzyme-based strategies to mitigate OTA contamination. In addition, from a general point of view, the findings indicate the benefit of this integrated computational and experimental workflow for the early identification of mycotoxin degrading enzymes. Indeed, this approach can be extended straightforwardly and systematically to other molecules, in order to broadly promote enzyme-based strategies to mitigate mycotoxin content along the food and feed production chain. Notably, our work proved that chemical similarities between a target mycotoxin and a set of already characterized ligands of specific enzymes, measured via target fishing studies, can be used as a consistent and probative feature for the purpose of identifying the degrading enzymes.

## 4. Materials and Methods

### 4.1. Assembly of the Ligands Database for Target Fishing

The three-dimensional (3D) structure of OTA was retrieved from PubChem (https://pubchem.ncbi.nlm.nih.gov; PubChem CID: 442530, accessed on 17 December 2018) and it was used as the template to screen a database of ligands derived from all of the small molecules in the bound state with the macromolecules recorded into the RCSB PDB databank (https://www.rcsb.org) [35] (Figure 2). All of the structures of small molecules were organized in the so defined ligand repository and, for the benefit of the present work, all non-redundant 3D structures of ligands were downloaded from the Ligand Expo Download page (http://ligand-expo.rcsb.org) in the chemical table file format (sdf format; 26.868 ligands accessed on December 17, 2018). The set of ligands was used to build up a database for a ligand-based virtual screening while using the FLAP software (https://www.moldiscovery.com) [36]. The default software setting was used to create the database. Molecular weight, the number of hydrogen bond donors/acceptors, and the calculated LogP values were used to describe the anatomy of database (Figure 3). Specifically, the number of hydrogen bond donors/acceptors and molecular weight were calculated using FLAP, while DataWarrior software [37] was used to calculate the theoretical LogP (Figure 3).

### 4.2. Ligand-Based Virtual Screening

The whole ligands database was virtually screened using FLAP (https://www.moldiscovery.com) [36], selecting OTA as an external template (Figure 2). Five different rounds of virtual screening (VS) with diverse accuracy level settings were performed, as follows:

(i) VS-1, using the so defined “bit string” mode to speed up computation time. In addition, the “shape penalty” was allowed to promote the selection of molecules with shape analogies;
(ii) VS-2, using the so defined “quadruplet” mode with a normal accuracy level;
(iii) VS-3, using the “dissimilarity” penalty in addition to the setting of VS-2 to promote the selection of molecules with shape analogies;
(iv) VS-4, using the so defined “quadruplet” mode with a maximum accuracy level; and,
(v) VS-5, using the “dissimilarity” penalty in addition to the setting of VS-4 to promote the selection of molecules with shape analogies.

The outcome of VSs are recorded under different parameters, such as “distance”, “Global Sum” and “Global Product”, which were used in combination to order ligands in the rank of most to least similar to OTA. In particular, “distance” estimates the overall chemical difference between each ligand and OTA (the lower the score, the more similar to OTA), while “Global Sum” and “Global Product” provide the overall scores while considering the sum and the product, respectively, of the various parameters and their combination (the higher the score, the closer the chemical similarity of ligands to OTA).

### 4.3. Structure-Based Molecular Modeling

In the structure-based molecular modeling, a docking study followed by molecular dynamic simulation were applied on the most promising OTA hydrolyzing enzymes that were identified by VSs to investigate the OTA-enzyme interaction from a molecular/atomistic level (Figure 2).

In particular, the software GOLD (Genetic Optimization for Ligand Docking) was used to define reliable architectures of OTA binding within the binding sites of selected proteins, in agreement with previous studies [38,39]. The models for each enzyme were derived from the respective PDB structure, as reported in Table 1. Software setting, protocols, models, and ligands preparation were performed, as reported previously [25]. As an exception, the use of external scoring functions was omitted and the native GOLDScore was used. GOLDScore might estimate the capability of ligands to satisfy the pocket requirements (the higher the score, the better the fitting within the pocket), as previously described by Dellafiora and co-workers [25]. In addition, the placement of the amidic bond of OTA was geometrically restrained close to the catalytic site of each enzyme to speed up computing time and optimize the analysis of explorable space with the aim of identifying potential enzymes that catalyze hydrolysis of OTA into Phe and OTα. For each protein target, the best-scored docking pose was considered to represent the most likely OTA binding architecture and it served as an input for the molecular dynamic (MD) simulations. The capability to produce reliable binding poses of docking was checked by computing the respective crystallographic ligands for each target protein and comparing with the crystallographic pose of same ligand in the target protein (further details are reported in the Supporting Material—Figures S1 to S18).

MD simulations were done to investigate the permanence of OTA within the binding site of protein targets along the time. The best binding pose that was calculated by docking simulation for each target was used as input for MD using GROMACS version 5.1.4 [40] with CHARMM27 all-atom force field parameters support [41]. OTA has been processed and parameterized with CHARMM27 all-atom force field using the SwissParam tool (http://www.swissparam.ch) [42]. Moreover, in case of incompletely resolved structures of target protein, a homology modeling approach using Modeller software version 9.19 [43] was applied before running MD to compute the missing parts, as described by Dellafiora [32]. Each protein-ligand (enzyme-OTA) complex was solvated with SPCE water in a cubic periodic boundary condition, and counter ions (Na^+^ and Cl^−^) were added to neutralize the system. Prior to MD simulation, the systems were energetically minimized in order to avoid steric clashes and correct improper geometries using the steepest descent algorithm with a maximum of 5000 steps. Afterwards, all the systems underwent isothermal (300 K, coupling time 2 psec) and isobaric (1-bar, coupling time 2 psec) 100 psec simulations before running 20 nsec simulations (300 K with a coupling time of 0.1 psec and 1-bar with a coupling time of 2.0 psec).

### 4.4. Estimation of OTA Hydrolyzing Activity

Neprilysin (EC: 3.4.24.11; Cat no. SRP6450-10UG), Matrix metalloproteinase 12 (MMP-12; EC: 3.4.24.65; Cat no. M9695-10UG), Carboxypeptidase B (CPB; EC: 3.4.17.2; Cat no. C9584-1MG), Urokinase (EC: 3.4.21.73; Cat no. U4010-10KU), and beta-site APP cleaving enzyme 1 (BACE-1; EC: 3.4.23.46; Cat no. S4195-50UG) were purchased from Sigma. Neprilysin, CPB and Urokinase were reconstituted in 100 µL, 200 µL, and 200 µL phosphate buffered saline buffer pH 7.4, respectively. MMP-12 was diluted 1:10 in phosphate buffered saline buffer pH 7.4 to a final volume of 100 µL. All content of BACE-1 was used directly, as supplied by Sigma in 20 mM Hepes, pH 7.4, containing 125 mM sodium chloride. Enzymatic assay was set up in 200 µL of reaction mixture, which contained 50 µL of reconstituted enzyme preparation, 100 µL of 400 ng/mL Ochratoxin A (prepared in the respective reaction buffer), and 50 µL of reaction buffer. The final concentration of OTA in each reaction mixture was 200 ng/mL. For BACE-1, 100 mM MES pH 4.5 was used as reaction buffer and for the other tested enzymes 100 mM sodium phosphate buffer pH 7.5. The final amount of enzyme in the 200 µL reaction mixture was 5 µg for Neprilysin, 5 µg for MMP-12, 665 µg for CPB, 2500 IU (as specified by supplier) for Urokinase, and 50 µg of BACE-1. Each reaction mixture was incubated at 37 °C for 180 min. along with separate negative reaction control, which contains OTA 200 ng/mL without enzyme. The treatment time and temperature were was scaled to 180 minutes and 37 °C to provide conditions that are meaningful for a practical implementation of the method meant in this study. A separate reaction was performed with 2 mg of Carboxypeptidase A (Sigma Cat no. C9268-500UN) in 200 µL reaction mix as a positive control. Each reaction was stopped adding 200 µL of acetonitrile at stored for 10 min. at 4 °C followed by centrifugation at 20,000 g for 10 min. at 4 °C. Afterward, 2 µL of supernatant were analyzed by HPLC-MS (Agilent 1290 Infinity II coupled to Sciex QTRAP 6500+ ESI, column Phenomenex Kinetex 2.6µm EVO C18 100 Å, 150x2.1 mm) to determine the concentration of OTA and its hydrolyzed product OTα. Three transitions of OTA (m/z 402 → 358+167+211) and OTα (m/z 255 → 211+167+123) were monitored to detect each compound, the transitions m/z 402 → 167 and m/z 255 → 167 were used to quantify OTA and OTα, respectively. A gradient of eluent B (94.9% ACN, 5% water, 0.1% formic acid) from 20% between 0.0–0.25 min. then gradually increased to 100% between 0.25–2.0 min. and stay at 100% between 2.00-2.50 min then immediately reduced to 20% at 2.51 min to calibrate column till 3.0 min. was used, in respect to eluent A consisted of 94.9% water, 5% ACN, 0.1% formic acid. Analytical grade pure OTA (Cat no. 10000346) and OTα (Cat no. 10003675) were purchased from Romer Labs GmbH and used in the setting up OTA hydrolyzing assay (including negative reaction control), as well as used as standard in the range of 100 ng/mL to 0.195 ng/mL for an estimation of disappearance of OTA and the appearance of OTα in the enzymatic reaction sample by HPLC-MS quantification (Figure S19, Supporting Material). Each sample was analyzed in duplicate, with CV below 10%. In case CV was more than 10%, the whole analysis was repeated.

## Figures and Tables

**Table 1:** Final list of ligands and related proteins.

Ligand PDB ID	Protein PDB ID	Protein Name	Source Organism	Note
TI2/TIO	1QF0/1ZDP	Thermolysin *	Bacteria	a
CXA	4DJL	Carboxypeptidase T (CPT)	Bacteria	a
CXA/ING	1IY7	Carboxypeptidase A (CPA) *	Bovine	a
CXA	5J1Q	Carboxypeptidase B (CPB)	Pig	a
FC0	1BCR/1WHT	Serine Carboxypeptidase II (Ser-CP II)	Wheat	a
TIO	5V48	Neprilysin	Rabbit	a
9UP	4FUG	Urokinase	Human	a
46L	4Y3S	Endothiapepsin	Fungus	a
S61	4AZ0	Cathepsin A	Human	a
DSV	2K2G	Matrix metalloproteinase 12 (MMP-12)	Human	a
1CH/ZY4/192	2WF4/1W51/4I11	Beta-secretase 1 (BACE 1)	Human	a
BBL	1ESB	Pancreatic elastase	Pig	a
P28	2ROY	Transthyretin	Human	b
9NF	2WX0	Serum albumin **	Human	b
1HN	3NKT	Salicylate 1,2-dioxygenase	Bacteria	c
NPQ	5FJO	N-acyl amino acid racemase	Bacteria	c

Note: ^a^ indicates potential OTA hydrolyzing enzymes (Figure 1); ^b^ indicates potential OTA binding proteins; ^c^ indicates potentially non-OTA hydrolyzing enzymes, able to transform OTA using other mechanisms than the hydrolysis of amidic bond. * Thermolysin and Carboxypeptidase A known for OTA hydrolyzing activity [20,21,28]. ** Serum albumin known as OTA binder [29,30,31].

## Supplementary Materials

The following are available online at https://www.mdpi.com/2072-6651/12/4/258/s1, Figures S1–S18: Graphics of structure-based molecular modeling for selected enzymes. Figure S19: HPLC-MS chromatograms. Table S1: List of PDB identification codes of ligands and proteins identified in the virtual screening before the expert selection of entries. Table S2: Docking scores collected in this study.

## References

[1] Marin S., Ramos A.J., Cano-Sancho G., Sanchis V. (2013). Mycotoxins: Occurrence, toxicology, and exposure assessment. Food Chem. Toxicol..

[2] van der Merwe K.J., Steyn P.S., Fourie L., Scott D.B., Theron J.J. (1965). Ochratoxin A, a toxic metabolite produced by Aspergillus ochraceus Wilh. Nature.

[3] Wu Q.H., Dohnal V., Huang L.L., Kuca K., Wang X., Chen G.Y., Yuan Z.H. (2011). Metabolic Pathways of Ochratoxin A. Curr. Drug Metab..

[4] Gan F., Zhou Y.J., Hou L.L., Qian G., Chen X.X., Huang K.H. (2017). Ochratoxin A induces nephrotoxicity and immunotoxicity through different MAPK signaling pathways in PK15 cells and porcine primary splenocytes. Chemosphere.

[5] Wu T.S., Lin Y.T., Huang Y.T., Yu F.Y., Liu B.H. (2020). Ochratoxin A triggered intracerebral hemorrhage in embryonic zebrafish: Involvement of microRNA-731 and prolactin receptor. Chemosphere.

[6] IARC (2012). Monographs on the Evaluation of Carcinogenic Risks to Humans: Chemical Agents and Related Occupations. A Review of Human Carcinogens. Lyon Fr..

[7] Denli M., Perez J.F. (2010). Ochratoxins in Feed, a Risk for Animal and Human Health: Control Strategies. Toxins.

[8] Becker-Algeri T.A., Castagnaro D., de Bortoli K., de Souza C., Drunkler D.A., Badiale-Furlong E. (2016). Mycotoxins in Bovine Milk and Dairy Products: A Review. J. Food Sci..

[9] Persi N., Pleadin J., Kovacevic D., Scortichini G., Milone S. (2014). Ochratoxin A in raw materials and cooked meat products made from OTA-treated pigs. Meat Sci..

[10] The European Commission (2006). Commission Regulation (EC) No 1881/2006 of 19 December 2006 setting maximum levels for certain contaminants in foodstuff. Off. J. Eur. Union.

[11] The European Commission (2006). Commission Recommendation (EU) 2016/1319 of 29 July 2016 amending recommendation 2006/576EC as regards deoxynivalenol, zearalenone and ochratoxin A in pet food. Off. J. Eur. Union.

[12] Karlovsky P., Suman M., Berthiller F., De Meester J., Eisenbrand G., Perrin I., Oswald I.P., Speijers G., Chiodini A., Recker T. (2016). Impact of food processing and detoxification treatments on mycotoxin contamination. Mycotoxin Res..

[13] Hassan Y.I., Zhou T. (2018). Promising Detoxification Strategies to Mitigate Mycotoxins in Food and Feed. Toxins.

[14] Varga J., Kocsube S., Peteri Z., Vagvolgyi C., Toth B. (2010). Chemical, Physical and Biological Approaches to Prevent Ochratoxin Induced Toxicoses in Humans and Animals. Toxins.

[15] Chen W.Y., Li C., Zhang B.Y., Zhou Z., Shen Y.B., Liao X., Yang J.Y.Q., Wang Y., Li X.H., Li Y.Z. (2018). Advances in Biodetoxification of Ochratoxin A-A Review of the Past Five Decades. Front. Microbiol..

[16] Dellafiora L., Galaverna G., Reverberi M., Dall’Asta C. (2017). Degradation of Aflatoxins by Means of Laccases from Trametes versicolor: An In Silico Insight. Toxins (Basel).

[17] Heinl S., Hartinger D., Thamhesl M., Vekiru E., Krska R., Schatzmayr G., Moll W.D., Grabherr R. (2010). Degradation of fumonisin B1 by the consecutive action of two bacterial enzymes. J. Biotechnol..

[18] Utermark J., Karlovsky P. (2007). Role of zearalenone lactonase in protection of Gliocladium roseum from fungitoxic effects of the mycotoxin zearalenone. Appl. Environ. Microbiol..

[19] Grenier B., Schwartz-Zimmermann H.E., Gruber-Dorninger C., Dohnal I., Aleschko M., Schatzmayr G., Moll W.D., Applegate T.J. (2017). Enzymatic hydrolysis of fumonisins in the gastrointestinal tract of broiler chickens. Poult. Sci..

[20] Pitout M.J. (1969). The hydrolysis of ochratoxin A by some proteolytic enzymes. Biochem. Pharmacol..

[21] Abrunhosa L., Paterson R.R.M., Venancio A. (2010). Biodegradation of Ochratoxin A for Food and Feed Decontamination. Toxins.

[22] Chang X.J., Wu Z.D., Wu S.L., Dai Y.S., Sun C.P. (2015). Degradation of ochratoxin A by Bacillus amyloliquefaciens ASAG1. Food Addit. Contam. A.

[23] Dobritzsch D., Wang H.M., Schneider G., Yu S. (2014). Structural and functional characterization of ochratoxinase, a novel mycotoxin-degrading enzyme. Biochem. J..

[24] Haq M., Gonzalez N., Mintz K., Jaja-Chimedza A., De Jesus C.L., Lydon C., Welch A.Z., Berry J.P. (2016). Teratogenicity of Ochratoxin A and the Degradation Product, Ochratoxin a, in the Zebrafish (Danio rerio) Embryo Model of Vertebrate Development. Toxins.

[25] Dellafiora L., Aichinger G., Geib E., Sánchez-Barrionuevo L., Brock M., Cánovas D., Dall’Asta C., Marko D. (2019). Hybrid in silico/in vitro target fishing to assign function to “orphan” compounds of food origin–The case of the fungal metabolite atromentin. Food Chem..

[26] Dellafiora L., Mena P., Del Rio D., Cozzini P. (2014). Modeling the effect of phase II conjugations on topoisomerase I poisoning: Pilot study with luteolin and quercetin. J. Agr. Food Chem..

[27] McKinney J.D., Richard A., Waller C., Newman M.C., Gerberick F. (2000). The practice of structure activity relationships (SAR) in toxicology. Toxicol. Sci..

[28] Dridi F., Marrakchi M., Gargouri M., Saulnier J., Jaffrezic-Renault N., Lagarde F. (2015). Comparison of carboxypeptidase Y and thermolysin for ochratoxin A electrochemical biosensing. Anal. Methods.

[29] Uchiyama S., Saito Y. (1987). Protein-binding potential of ochratoxin A in vitro and its fluorescence enhancement. J. Food Hyg. Soc. Japan.

[30] Koszegi T., Poor M. (2016). Ochratoxin A: Molecular Interactions, Mechanisms of Toxicity and Prevention at the Molecular Level. Toxins.

[31] Il’ichev Y.V., Perry J.L., Simon J.D. (2002). Interaction of ochratoxin A with human serum albumin. Preferential bining of the dianion and pH effects. J. Phys. Chem. B.

[32] Dellafiora L., Galaverna G., Cruciani G., Dall’Asta C. (2019). A computational study toward the “personalized” activity of alternariol - Does it matter for safe food at individual level?. Food Chem. Toxicol..

[33] Abrunhosa L., Santos L., Venancio A. (2006). Degradation of ochratoxin A by proteases and by a crude enzyme of Aspergillus niger. Food Biotechnol..

[34] Kolli N., Garman S.C. (2014). Proteolytic Activation of Human Cathepsin A. J. Biol. Chem..

[35] Consortium W. (2019). Protein Data Bank: The single global archive for 3D macromolecular structure data. Nucleic Acid Res..

[36] Baroni M., Cruciani G., Sciabola S., Perruccio F., Mason J.S. (2007). A common reference framework for analyzing/comparing proteins and ligands. Fingerprints for Ligands and Proteins (FLAP): Theory and application. J. Chem. Inf. Model..

[37] Sander T., Freyss J., von Korff M., Rufener C. (2015). DataWarrior: An open-source program for chemistry aware data visualization and analysis. J. Chem. Inf. Model..

[38] Dellafiora L., Galaverna G., Dall’Asta C., Cozzini P. (2015). Hazard identification of cis/trans-zearalenone through the looking-glass. Food Chem. Toxicol..

[39] Maldonado-Rojas W., Olivero-Verbel J. (2011). Potential interaction of natural dietary bioactive compounds with COX-2. J. Mol. Graph. Model..

[40] Abraham M.J., Murtola T., Schulz R., Páll S., Smith J.C., Hess B., Lindahl E. (2015). GROMACS: High performance molecular simulations through multi-level parallelism from laptops to supercomputers. SoftwareX.

[41] Best R.B., Zhu X., Shim J., Lopes P.E., Mittal J., Feig M., Mackerell A.D.J. (2012). Optimization of the additive CHARMM all-atom protein force field targeting improved sampling of the backbone φ, ψ and side-chain χ(1) and χ(2) dihedral angles. J. Chem. Theory Comput..

[42] Zoete V., Cuendet M.A., Grosdidier A., Michielin O. (2011). SwissParam: A Fast Force Field Generation Tool for Small Organic Molecules. J. Comput. Chem..

[43] Webb B., Sali A. (2016). Comparative Protein Structure Modeling Using Modeller. Curr. Protoc. Bioinformatics.

