# Supplementary material for: An In Silico Target Fishing Approach to Identify Novel Ochratoxin A Hydrolyzing Enzyme
Source: Toxins (Basel). 2020 Apr 16;12(4):258. doi: 10.3390/toxins12040258 (PMC7232302; doi:10.3390/toxins12040258)
Supplement: Supplementary file 1 [file toxins-12-00258-s001.pdf]

Supplementary Materials: An In Silico Target Fishing Approach to Identify Novel Ochratoxin A Hydrolyzing Enzyme

Luca Dellafiora, Christoph Gonaus, Barbara Streit, Gianni Galaverna, Wulf-Dieter Moll, Gudrun Vogtentanz, Gerd Schatzmayr, Chiara Dall'Asta and Shreenath Prasad

Graphics of Structure-Based Molecular Modeling

The graphics of the most promising results from structure-based molecular modeling are reported below. Table S2 reports all of the computed docking scores.

Carboxypeptidase T (CPT)

CPT is a bacterial carboxypeptidase homologous to the bovine carboxypeptidase A (CPA). Both enzymes are recorded in the Protein Databank (PDB) in the bound state with CXA (phenylalnine-N-sulphonate), one of the OTA-like compounds identified in the ligand-based virtual screening. Therefore, the pose of CXA was computed in both enzymes (CPT and CPA) and it was then compared to the respective crystallographic pose to assess the reliability of docking procedure on this model. The score recorded for CXA within CPA and CPT was 95.8 and 96.1, respectively. Docking poses were in stark agreement with crystallographic data pointing to the model reliability (Figure S1A and S1B). Subsequently, the interaction between OTA and CPA was calculated and compared with the calculated pose of OTA within carboxypeptidase T. The scores that were recorded for OTA within CPT and CPA were 95.7 and 100.1, respectively, pointing to the capability of OTA to well satisfy the requirements of both pockets. However, a quantitative comparison could not be done due to the lack of complete training and validation. Nevertheless, OTA showed a comparable pocket arrangement in the two enzymes, further supporting its substrate-like interaction, as shown in Figure S1C.

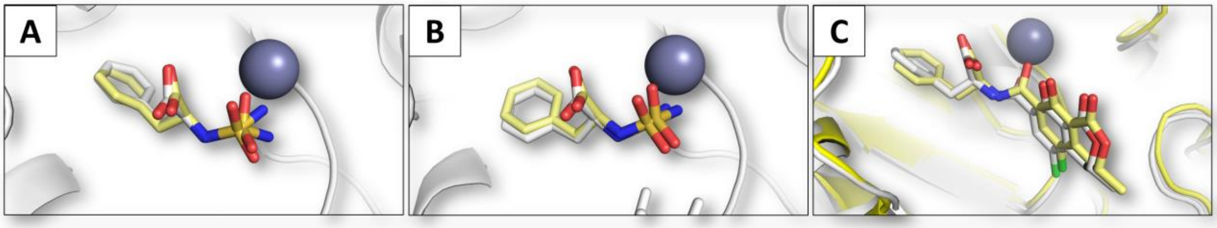

Figure S1. Docking results of carboxypeptidases (Carboxypeptidase T (CPT) and carboxypeptidase A (CPA)). Proteins are represented in cartoon, ligands in sticks and Zn2+ ions in spheres. (**A**) Comparison between the calculated (yellow) and crystallographic (white) pose of CXA within CPA (PDB ID: 1IY7). (**B**) Comparison between the calculated (yellow) and crystallographic (white) pose of CXA within CPT (PDB ID: 4DJL). (**C**) Comparison between the calculated pose of OTA within CPA (white; model derived from Protein Databank (PDB) structure having code 1IY7) and CPT (yellow). .

Subsequently, the complex underwent MD simulations to check its geometrical stability. Both OTA and CPT showed a marked geometrical stability throughout the simulation, as shown in Figure S2A and S2B. In addition, the analysis of OTA trajectory revealed its persistence within the catalytic site stably adopting an orientation likely suitable to undergo the reaction of OTA hydrolysis into OTα and Phe (Figure S2C, Figure 2).

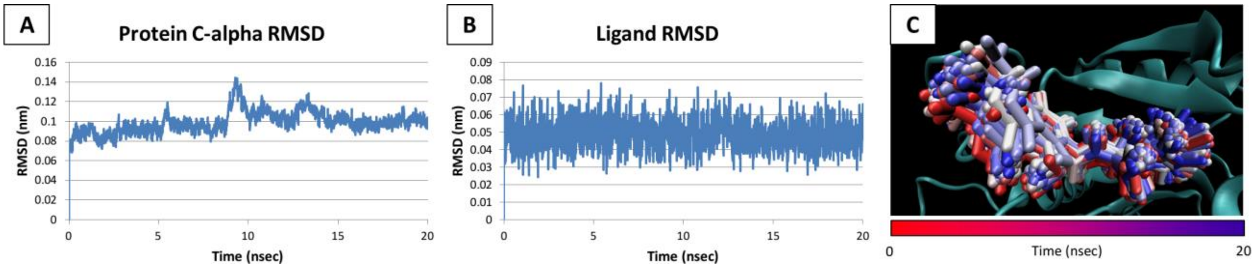

Figure S2. Molecular dynamics results of CPT-OTA complex. (**A**) RMSD plot of CPT in complex with OTA. (**B**) RMSD plot of OTA in complex with CPT. (**C**) Time-step representation of the OTA trajectory. The color switch from-red-to-blue indicates the stepwise changes of ligand coordinates along the 20nsec simulation.

Carboxypeptidase B (CPB)

CPB is a porcine homologous of the bovine carboxypeptidase A (CPA). Similarly to CPT, this enzyme is recorded in PDB in complex with CXA (phenylalanine-N-sulphonate). Therefore, CXA was docked and its pose was compared to its crystallographic architecture of binding in order to assess the model reliability. CXA scored 84.7 GoldScore units. The calculated pose of CXA was in strong agreement with its crystallographic architecture supporting, also in this case, the model reliability, as shown in Figure S3A. Concerning OTA, it scored 95.3 GOLDScore units, suggesting its capability to well satisfy the pocket, as shown within CPA and CPT. In addition, the computed pose of OTA within CPB was compared with the one calculated within CPA. OTA showed the same mode of binding in both enzymes (Figure S3B), further supporting its substrate-like interaction within this enzyme.

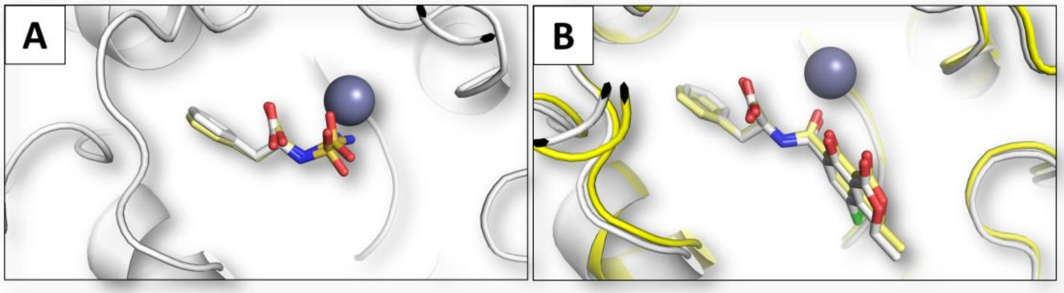

Figure S3. Docking results of CPB. Proteins are represented in cartoon, ligands in sticks and Zn2+ ions in spheres. (**A**) Comparison between the calculated (yellow) and crystallographic (white) pose of CXA within CPB (PDB ID: 5J1Q). (**B**) Comparison between the calculated pose of OTA within CPA (white) and CPB (yellow). .

Subsequently, the OTA-CPB complex underwent MD simulations to check the geometrical stability. The protein was found to be stable throughout the simulation, while OTA showed an early change of geometry, which was kept stable up to the end of simulation (Figure S4A and S4B). Notably, the analysis of OTA trajectory excluded the existence of outward trajectories. In addition, the thorough analysis of OTA movements pointed out that the coumarin-like moiety was mainly reorganized during the simulation, while the amidic bond and Phe-part were both kept stable and theoretically well organized with respect to Zn ion to undergo the reaction of OTA hydrolysis into OTα and Phe (Figure S4C, Figure 2).

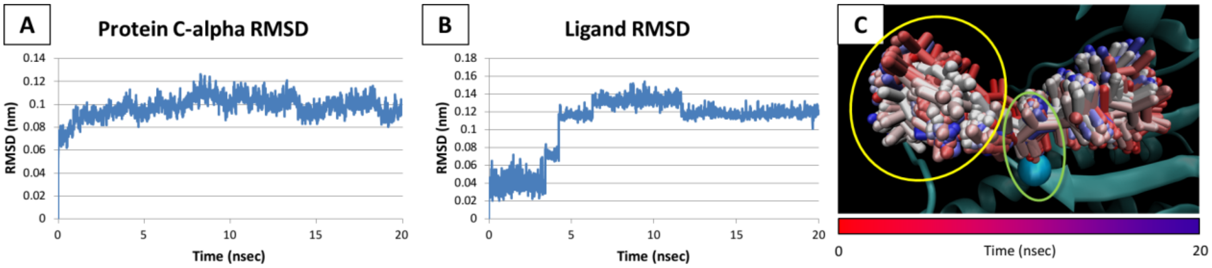

Figure S4. Molecular dynamics results of CPB-OTA complex. (**A**) RMSD plot of CPB in complex with OTA. (**B**) RMSD plot of OTA in complex with CPB. (**C**) Time-step representation of the OTA trajectory. The color switch from-red-to-blue indicates the stepwise changes of ligand coordinates along the 20 nsec simulation. The yellow ring indicates the coumarin-like moiety, while the green ring indicates the localization of Zn ion (represented by sphere) and OTA amidic bond.

Serine Carboxypeptidase II (Ser-CP II)

Ser-CP II is a serine-carboxypeptidase from wheat related to the already known OTA-hydrolyzing enzyme Carboxypeptidase Y (CPY) from bakery yeast. The Ser-CP II is recorded in PDB in the bound state with FC0 (N-carboxy-L-phenylalanine), one of the OTA-like compounds identified in the ligand-based virtual screening. FC0 was docked and its pose was compared to its crystallographic architecture of binding to assess the reliability of docking procedure on this model. The score recorded for FC0 was 57.5. The calculated and crystallographic poses were in strong agreement, suggesting the model reliability, as shown in Figure S5A. Subsequently, the interaction between OTA and Ser-CP II was calculated and compared with the calculated pose of OTA within CPY. The scores recorded for OTA within the Ser-CP II and CPY were 54.7 and 73.4, respectively, pointing to the capability of OTA to satisfy the requirements of both pockets well. However, the interaction with the latter could not be considered more favorite, in spite of the higher score recorded, due to the lack of complete training and validation procedures. Concerning the architectures of binding, OTA showed a slight different organization due to the Thr60Tyr substitution in the binding pocket of Ser-CP II that caused a different organization of the Phe moiety of OTA (Figure 5B).

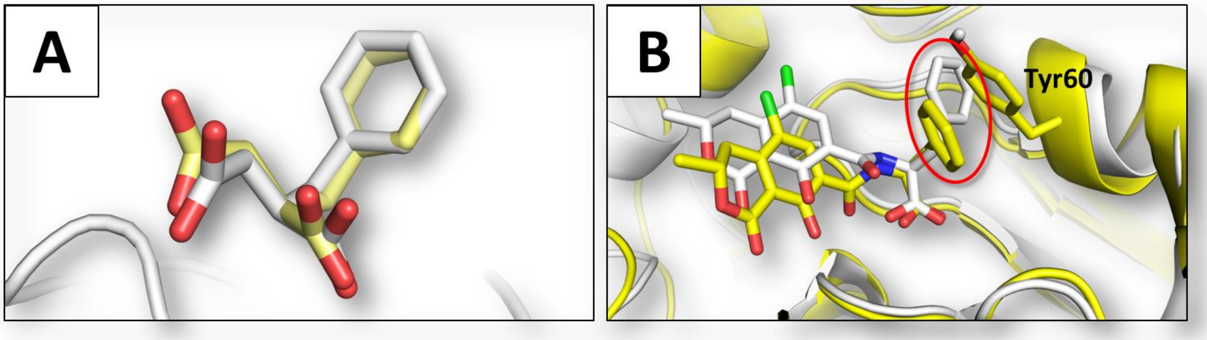

Figure S5. Docking results of Ser-CP II. Proteins are represented in cartoon, while ligands in stick. A. Comparison between the calculated (yellow) and crystallographic (white) pose of FC0 within the Ser-CP II (PDB ID 1WHT). B. Comparison between the calculated pose of OTA within CPY (white; model derived from PDB structure having code 1YSC) and the Ser-CP II (yellow). The red ring indicates the reorganization of Phe moiety within the two enzymes.

Subsequently, the OTA-serine carboxypeptidase II complex underwent MD simulations to check its geometrical stability. The protein was found stable throughout the simulation, while OTA was found to be more mobile (Figure S6A and S6B). In addition, the analysis of OTA trajectory revealed a marked trajectory outward the inner part of catalytic site (Figure S6C).

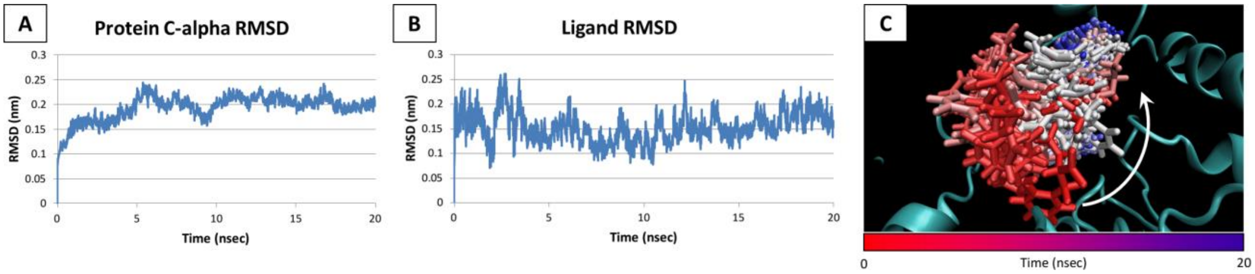

Figure S6. Molecular dynamics results of Ser-CP II-OTA complex. (**A**) RMSD plot of the enzyme in complex with OTA. (**B**) RMSD plot of OTA in complex with Ser-CP II. (**C**) Time-step representation of the OTA trajectory. The color switch from-red-to-blue indicates the stepwise changes of ligand coordinates along the 20 nsec simulation. The white arrow indicates the outward trajectory.

Neprilysin

Neprilysin is a Zn-dependent endopeptidase that is able to hydrolyze peptides up to 50 residues. This enzyme is recorded in PDB in the bound state with TIO [thiorphan: (2-mercaptomethyl-3-phenyl-propionyl)-glycine], one of the OTA-like compounds identified in the ligand-based virtual screening. Therefore, TIO was docked and its pose was compared to its crystallographic architecture of binding in order to assess the reliability of docking procedure on this model. The score recorded for TIO was 64.5. The calculated and crystallographic poses were in strong agreement, suggesting the model reliability, as shown in Figure S7A. Subsequently, the interaction of OTA was calculated, recording a score of 87.9 units. The Phe portion of OTA superimposed the benzene moiety of thiorphan orienting the amide bond to the Zn ion in a reasonably proper position to undergo hydrolysis, as shown in Figure S7B.

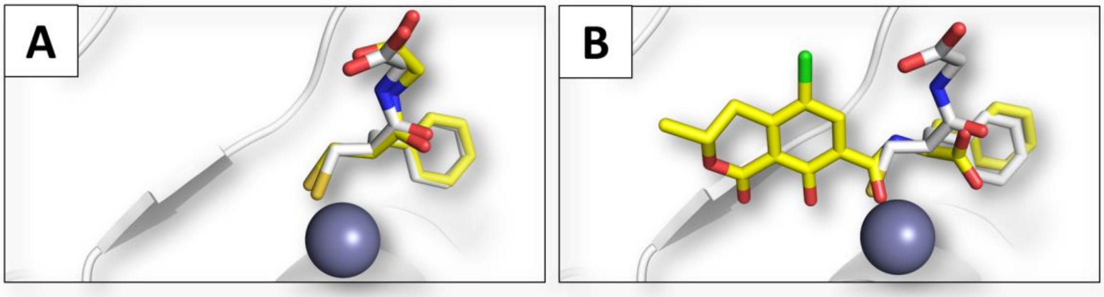

Figure S7. Docking results of neprilysin-OTA complex. The protein is represented in cartoon, while ligands in stick. The sphere represents the Zn ion. (**A**) Comparison between the calculated (yellow) and crystallographic (white) pose of TIO within rabbit neprilysin (PDB ID 5V48). (**B**) Calculated pose of OTA (yellow) superimposed to the crystallographic pose of thiorphan (while; PDB ID 5V48).

Subsequently, the OTA-neprylisin complex underwent molecular dynamic simulations to check its geometrical stability. The protein was found stable throughout the simulation while OTA was found to be more mobile (Figure S8A and S8B). In particular, the analysis of trajectories revealed that OTA had an early increase of RMSD, mainly due to the reorganization of the coumarin-like moiety, but the overall organization of the amidic bound in respect to the Zn ion was kept stable throughout the simulation (Figure S8C).

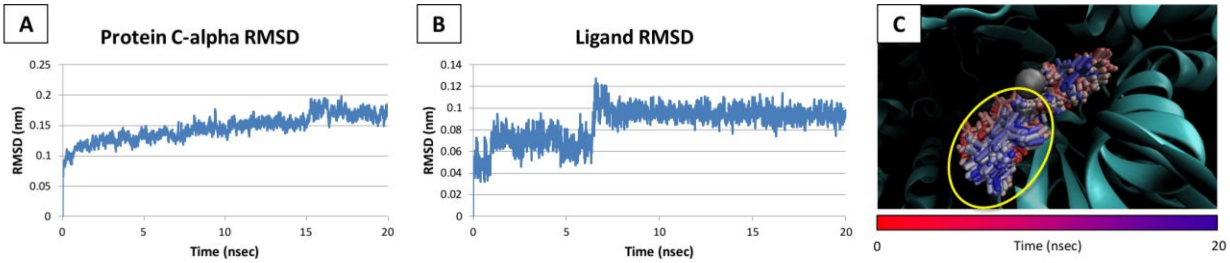

Figure S8. Molecular dynamics results of neprilysin-OTA complex. (**A**) RMSD plot of neprilysin in complex with OTA. (**B**) RMSD plot of OTA in complex with neprilysin. (**C**) Time-step representation of the OTA trajectory. The color switch from-red-to-blue indicates the stepwise changes of ligand coordinates along the 20nsec simulation. The yellow ring indicates the coumarin-like moiety. The Zn ion is represented by sphere.

Urokinase

Urokinase is a serine protease that is related to the already characterized OTA hydrolyzing enzyme bovine alpha chymotrypsin. This enzyme is recorded in PDB in the bound state with 9UP [methyl(7-carbamimidoylnaphthalen-1-yl)carbamate], one of the OTA-like compounds identified in the ligand-based virtual screening. Therefore, the computed pose of 9UP was compared to its crystallographic architecture of binding to assess the reliability of docking procedure on this model. The score that was recorded for 9UP was 56.0. The calculated and crystallographic poses were in strong agreement suggesting the model reliability, as shown in Figure S9A. Subsequently, the interaction of OTA was calculated, recording a score of 64.3 units. The coumarin-like moiety of OTA superimposed the aromatic portion of 9UP, while the amide bond was in a reasonably well-oriented position in respect to the catalytic serine to undergo hydrolysis, as shown in Figure S9B.

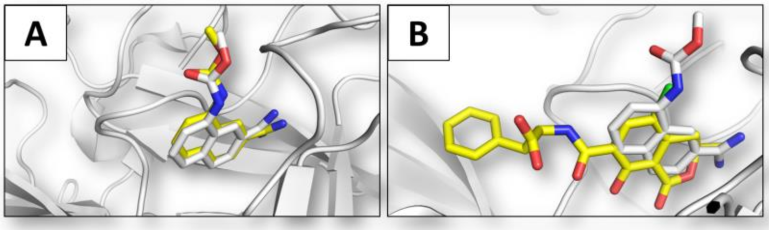

Figure S9. Docking results of urokinase-OTA complex. The protein is represented in cartoon, while ligands in stick. (**A**) Comparison between the calculated (yellow) and crystallographic (white) pose of 9UP within human urokinase (PDB ID 4FUG). (**B**) Calculated pose of OTA (yellow) superimposed to the crystallographic pose of 9UP (white; PDB ID 4FUG).

Subsequently, the OTA-urokinase complex underwent molecular dynamic simulations to check its geometrical stability. The protein was found to be stable throughout the simulation, while OTA was found more mobile (Figure S10A and S10B). In particular, the analysis of trajectories revealed that the Phe moiety was the most mobile, while the coumarin-like moiety was found to be quite stable along the all simulation (Figure S10C). Nevertheless, OTA was found to be stably buried within the catalytic cleft during the all simulation. Notably, the mobility of Phe moiety started to increase early (nearly after 3 nsec of simulation) and tended to get stabilized again similarly to the starting pose toward the end of simulation (in the last 5 nsec), as shown in Figure S10B.

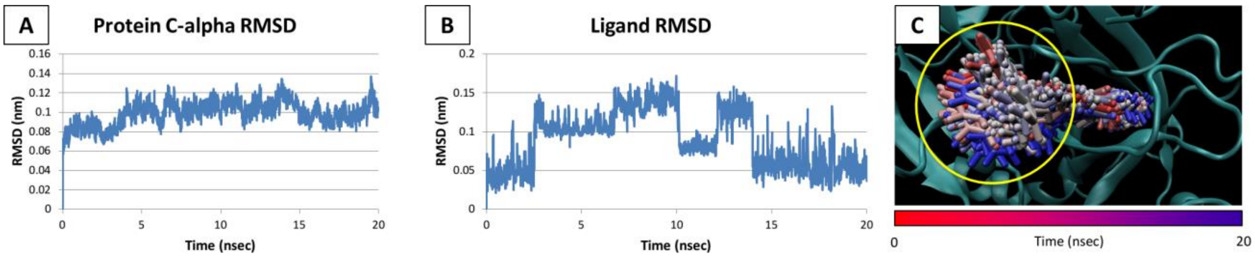

Figure S10. Molecular dynamics results of urokinase-OTA complex. (**A**) RMSD plot of urokinase in complex with OTA. (**B**) RMSD plot of OTA in complex with urokinase. (**C**) Time-step representation of the OTA trajectory. The color switch from-red-to-blue indicates the stepwise changes of ligand coordinates along the 20 nsec simulation. The yellow ring indicates the Phe moiety.

Endothiapepsin

Endothiapepsin is a pepsin-like aspartic peptidase. This enzyme is recorded PDB in the bound state with 46L [6-(dimethylamino)pyridine-3-carboxylic acid], one of the OTA-like compounds identified in the ligand-based virtual screening. Therefore, the computed pose of 46L was compared to its crystallographic architecture of binding to assess the reliability of docking procedure on this model. The score recorded for 46L was 35.4. The calculated and crystallographic poses were in strong agreement suggesting the model reliability, as shown in Figure S11A. Subsequently, the interaction of OTA was calculated, recording a score of 62.4 units. OTA adopted an organization that was markedly different from the one of 46L, as shown in Figure S11A. Nevertheless, the amidic bond was placed in correspondence to the catalytic core of the enzyme adopting a position theoretically suitable to undergoing hydrolysis.

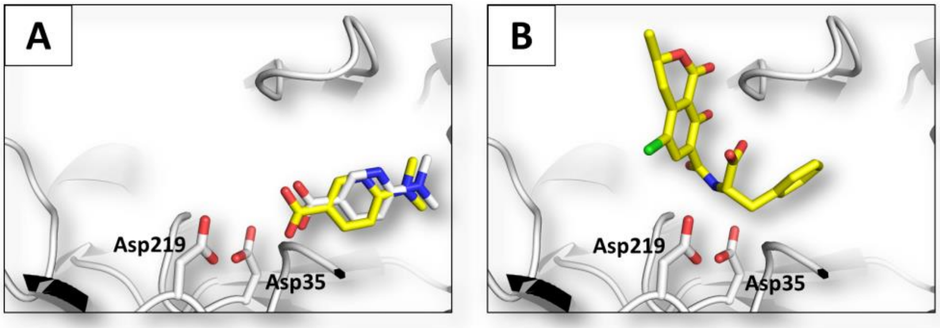

Figure S11. Docking results of endothiapepsin-OTA complex. The protein is represented in cartoon, while ligands in sticks. (**A**) Comparison between the calculated (yellow) and crystallographic (white) pose of 46L within fungal endothiapepsin (PDB ID 4Y3S). (**B**) Calculated pose of OTA (yellow).

Subsequently, the OTA-endothiapepsin complex underwent molecular dynamic simulations to check its geometrical stability. The protein and OTA both showed an overall geometrical instability, as marked RMSD variations were observed, as shown in Figure S12A and S12B. In addition, the analysis of OTA trajectories revealed a marked outward movement from the catalytic site.

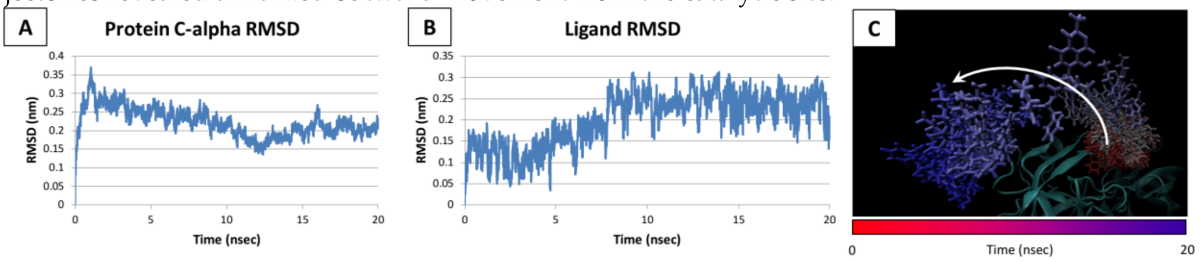

Figure S12. Molecular dynamics results of endothiapepsin-OTA complex. (**A**) RMSD plot of endothiapepsin in complex with OTA. (**B**) RMSD plot of OTA in complex with endothiapepsin. (**C**) Time-step representation of the OTA trajectory. The color switch from-red-to-blue indicates the stepwise changes of ligand coordinates along the 20nsec simulation. The white arrow indicates the outward trajectory of OTA from the catalytic site.

Cathepsin A

Cathepsin A is a broad-spectrum serine protease that is homologous to the already characterized OTA-hydrolyzing enzyme carboxypeptidase Y (CPY). This enzyme is recorded in PDB the bound state with S61: (S)-3-{[1-(2-fluro-phenyl)-5-hydroxy-1H-pyrazole-3-carbonyl]-amino}-3-o-tolyl-propionic acid, one of the OTA-like compounds identified in the ligand-based virtual screening. Therefore, the computed pose of S61 was compared to its crystallographic architecture of binding to assess the reliability of docking procedure on this model. The score recorded for S61 was 64.7. The calculated and crystallographic poses were in good agreement suggesting the model reliability, as shown in Figure S13A. Subsequently, the interaction of OTA was calculated, recording a score of 59.6 units. OTA adopted a binding pose resembling the one observed within the wheat serine carboxypeptidase II (Ser-CP II; see above) and arranging the amidic bond in a possibly suitable orientation to undergo hydrolysis, as shown in Figure S13B.

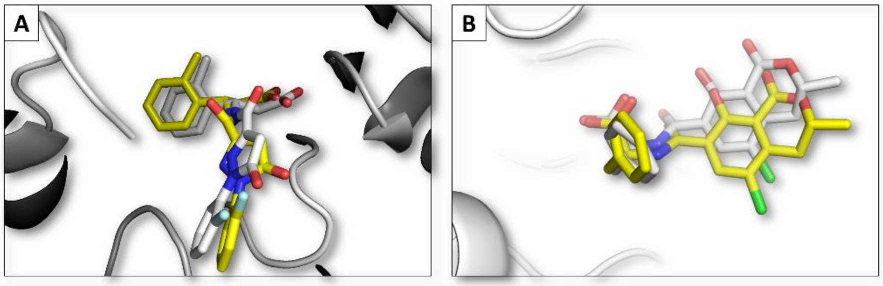

Figure S13. Docking results of cathepsin A-OTA complex. The protein is represented in cartoon, while ligands in stick. (**A**) Comparison between the calculated (yellow) and crystallographic (white) pose of S61 within human cathepsin A (PDB ID 4AZ0). (**B**) Calculated poses of OTA within human cathepsin A (yellow) and Ser-CP II (white).

Subsequently, the OTA-cathepsin A complex underwent MD simulations to check its geometrical stability. Protein and OTA showed an increasing mobility trend along the considered timeframe, though of slight intensity, as shown in Figure S14. In addition, the analysis of OTA trajectory did not show a geometrically stable interaction with the site. This might be due to the incompleteness of the model used (seven residues close to the OTA binding site were missing).

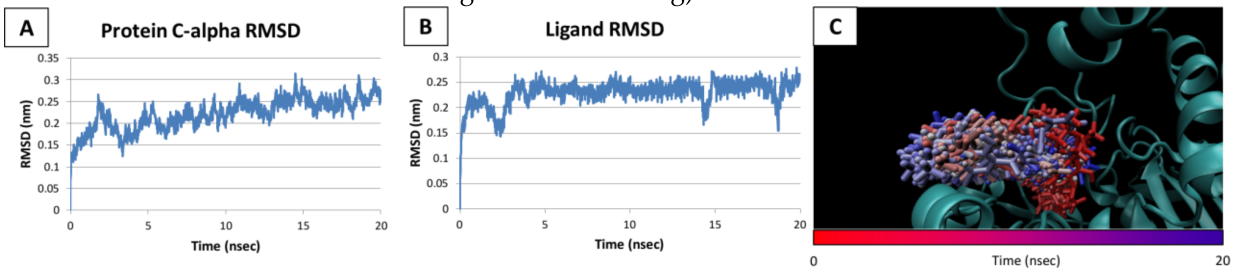

Figure S14. Molecular dynamics results of cathepsin A-OTA complex. (**A**) RMSD plot of cathepsin A in complex with OTA. (**B**) RMSD plot of OTA in complex with cathepsin A. (**C**) Time-step representation of the OTA trajectory. The color switch from-red-to-blue indicates the stepwise changes of ligand coordinates along the 20nsec simulation.

Matrix Metalloproteinase 12 (MMP-12)

MMP-12 is a Zn- and Ca-dependent protease that cleaves a broad range of substrates. This enzyme is recorded in PDB in the bound state with DSV: N-(dibenzo[b,d]thiophen-3-ylsulfonyl)-L-valine, one of the OTA-like compounds identified in the ligand-based virtual screening. Therefore, the computed pose of DSV was compared to its crystallographic architecture of binding to assess the reliability of docking procedure on this model. The score recorded for DSV was 83.4. The calculated and crystallographic poses were in strong agreement, suggesting the model reliability, as shown in Figure S15A. Subsequently, the interaction of OTA was calculated, recording a score of 59.7 units. The superimposition of the calculated pose of OTA with the crystallographic pose of DSV revealed that the carboxylate of OTA arranged similarly to the sulphonyl group of DSV, rather than mimicking its carboxylate group, as shown in figure S15B.

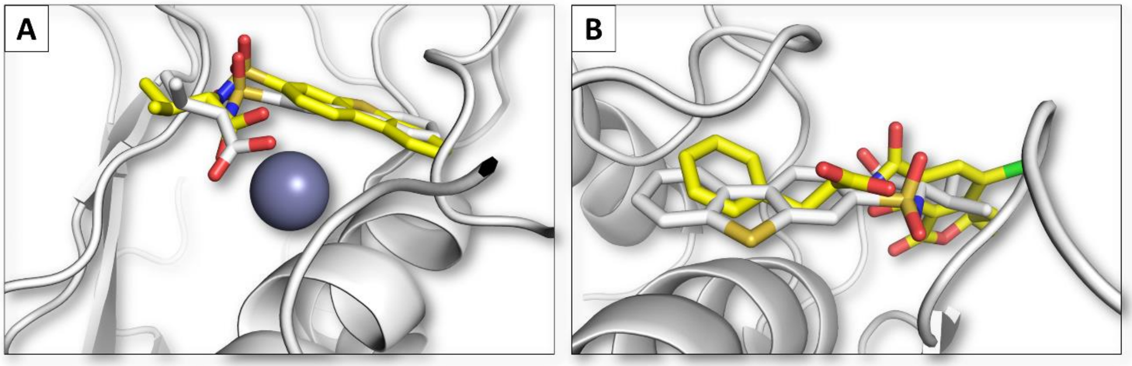

Figure S15. Docking results of MMP12-OTA complex. The protein is represented in cartoon, while ligands in stick. Zn ion is represented by sphere. (**A**) Comparison between the calculated (yellow) and crystallographic (white) pose of DSV within human MMP-12 (PDB ID 2K2G). (**B**) Calculated poses of OTA within human MMP-12 (yellow) in comparison to the crystallographic pose of DSV.

Subsequently, the OTA-MMP12 complex underwent molecular dynamic simulations to check its geometrical stability. The protein and OTA were found overall stable along the simulation, as shown in Figure S16. In addition, the analysis of OTA trajectory revealed that the toxin stably persisted close to the catalytic site arranging the amidic bond in the proper orientation to undergo hydrolysis.

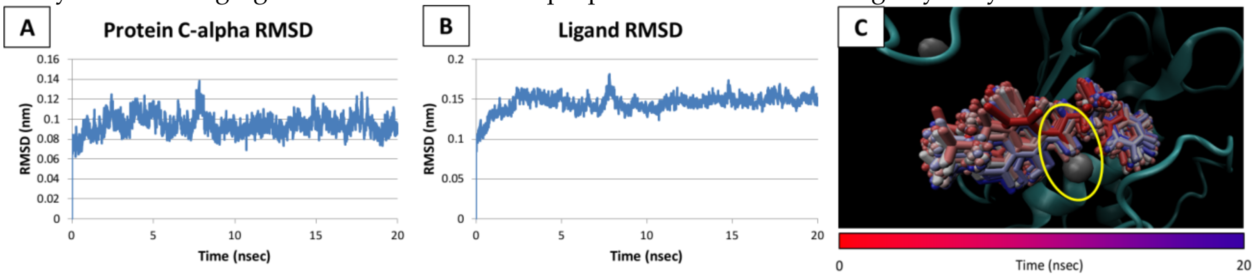

Figure S16. Molecular dynamics results of MMP12-OTA complex. (**A**) RMSD plot of MMP-12 in complex with OTA. (**B**) RMSD plot of OTA in complex with MMP-12. (**C**) Time-step representation of the OTA trajectory. The color switch from-red-to-blue indicates the stepwise changes of ligand coordinates along the 20 nsec simulation. The yellow ring indicates the position of Zn ion and amidic bond.

Beta-Secretase 1 (BACE1)

The enzyme is an endopeptidase with a relatively non-stringent specificity that is homologous to the aspartic proteases of the pepsin family. The protein is recorded in PDB in the bound state with ZY4, one of the OTA-like compounds identified in the ligand-based virtual screening. Therefore, the computed pose of ZY4 was compared to its crystallographic architecture of binding to assess the reliability of docking procedure on this model. The score recorded for 1HN was 99.9. The calculated and crystallographic poses were in strong agreement suggesting the model reliability, as shown in Figure S17A. Subsequently, the

interaction of OTA was calculated, recording a score of 58.2 units. Figure S17B illustrates OTA oriented the amidic bond towards the catalytic aspartates and the superimposition of its pose with the crystallographic pose of ZY4.

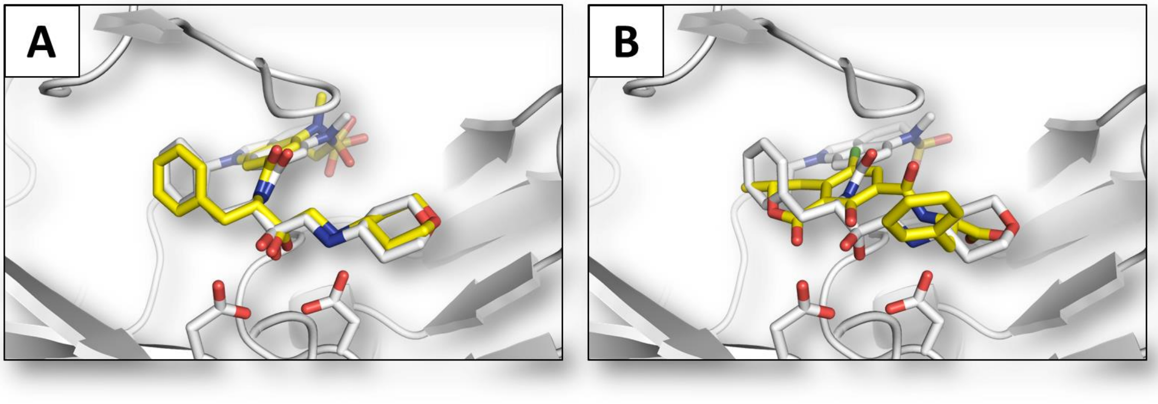

Figure S17. Docking results of OTA in complex with BACE1. The protein is represented in cartoon, while ligands in sticks. (**A**) Comparison between the calculated (yellow) and crystallographic (white) pose of ZY4 within BACE1 (PDB ID 2WF4) (**B**) Calculated poses of OTA (yellow) in comparison to the crystallographic pose of ZY4 (white).

Subsequently, the OTA-BACE1 complex underwent MD simulations to check its geometrical stability. Protein and OTA got stabilized toward the end of the simulation, as shown in Figure S18. In addition, the analysis of OTA trajectory revealed that the toxin described an outward movement followed by an inward migration toward the binding site in the last part of the simulation.

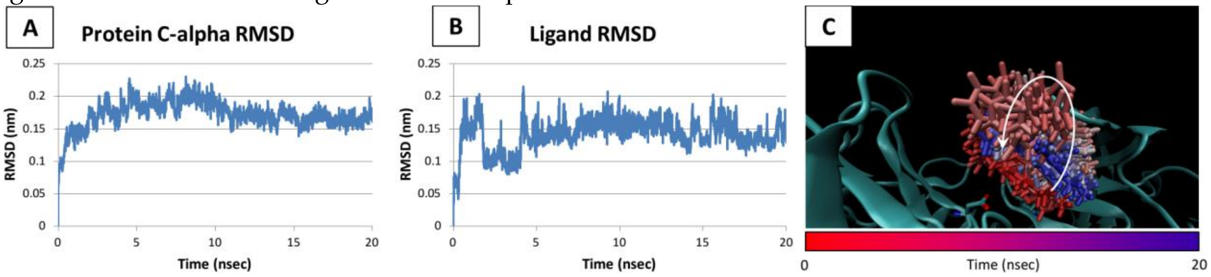

Figure S18. Molecular dynamics results of BACE1-OTA complex. (**A**) RMSD plot of BACE1 in complex with OTA. (**B**) RMSD plot of OTA in complex with BACE1. (**C**) Time-step representation of the OTA trajectory. The color switch from-red-to-blue indicates the stepwise changes of ligand coordinates along the 20 nsec simulation. The white arrow indicates the outward-inward trajectory of OTA.

HPLC-MS Chromatograms

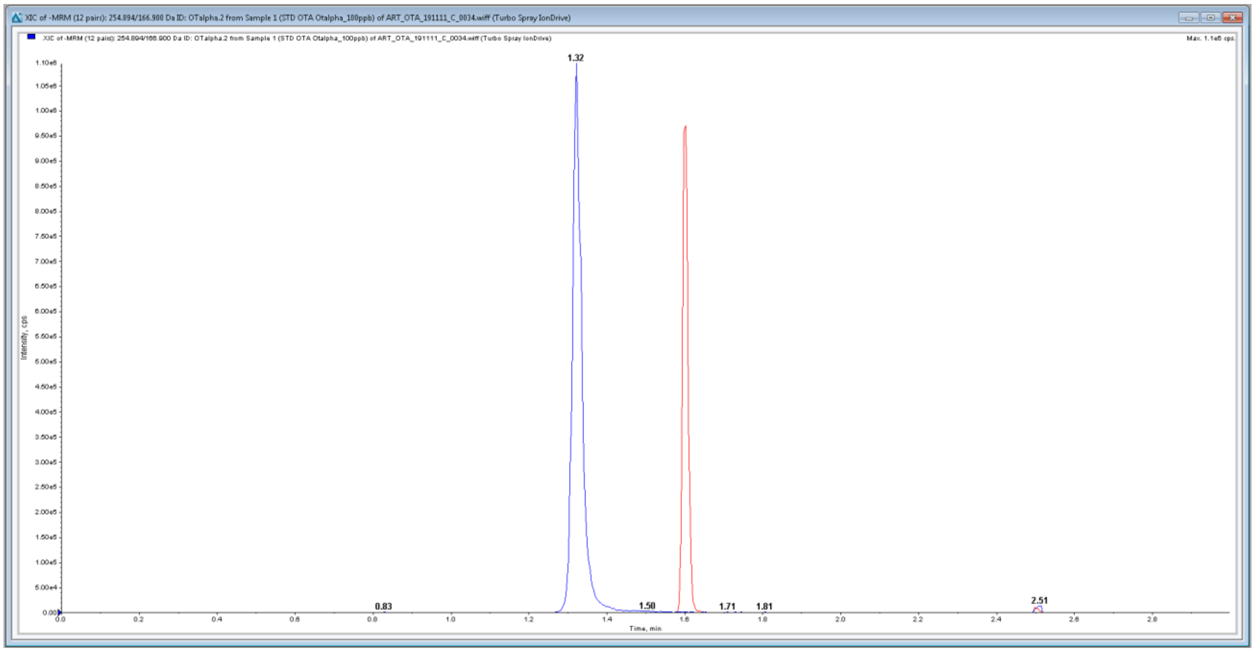

(**A**) HPLC-MS chromatogram of standard sample containing 100 ng/ml OTA (red peak) and 100ng/ml OT*α* (blue peak).

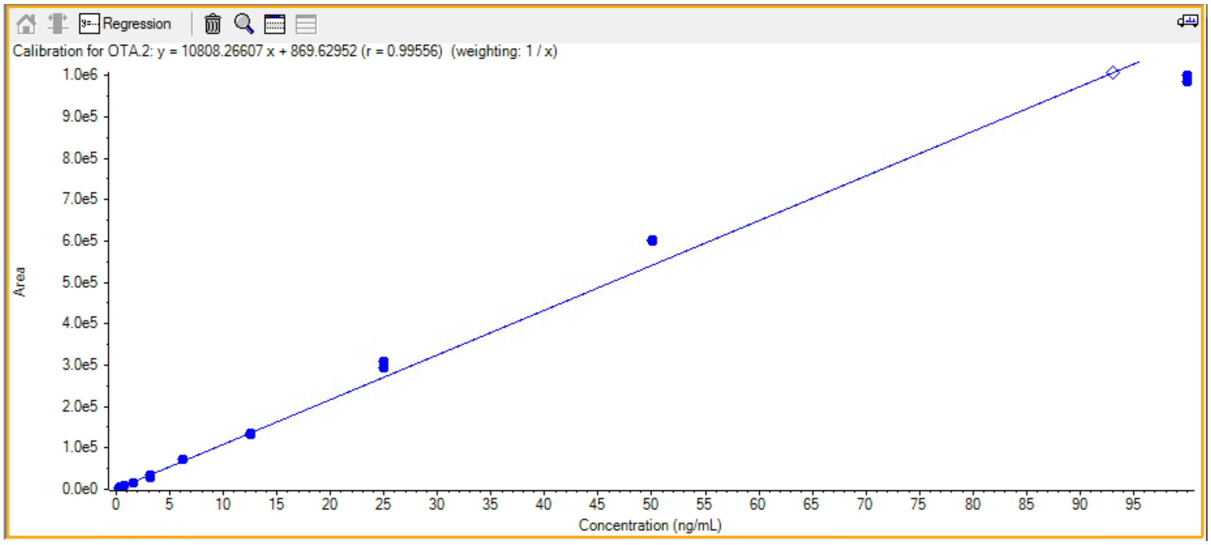

(**B**) HPLC-MS standard curve (peak area vs concentration) of OTA in the concentration range of 0.195 – 100 ng/ml (•). OTA measured (93.1 ng/ml) in CPB reaction sample is also plotted (◊). 96.0 ng/ml OTA was measured in negative control (without enzyme) instead of 100 ng/ml, which is not plotted in this curve. The difference of OTA measure in negative control and CBP reaction sample was 2.9 ng/ml i.e. the amount of OTA hydrolyzed into OT*α* due to enzymatic reaction of CPB, which is matching with OT*α* estimated in the CPB reaction sample (see below Figure S19C)

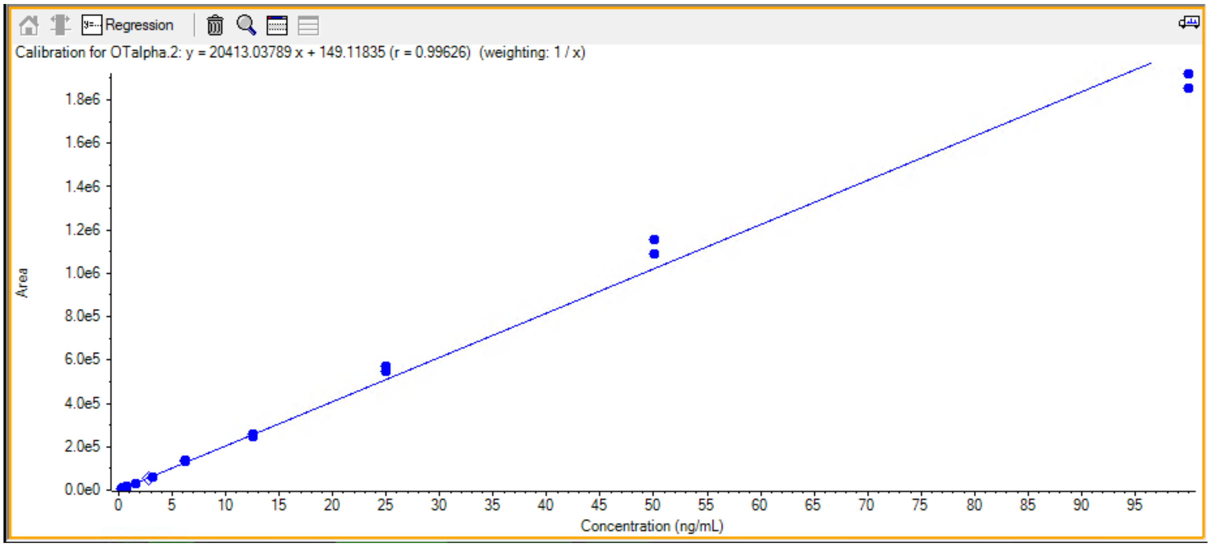

(**C**) HPLC-MS standard curve (peak area vs concentration) of OT*α* in the concentration range of 0.195 – 100 ng/ml (•). OT*α* measured (2.79 ng/ml) in CPB reaction sample is also plotted (◊). OT*α* was not at all measured in negative control (without enzyme).

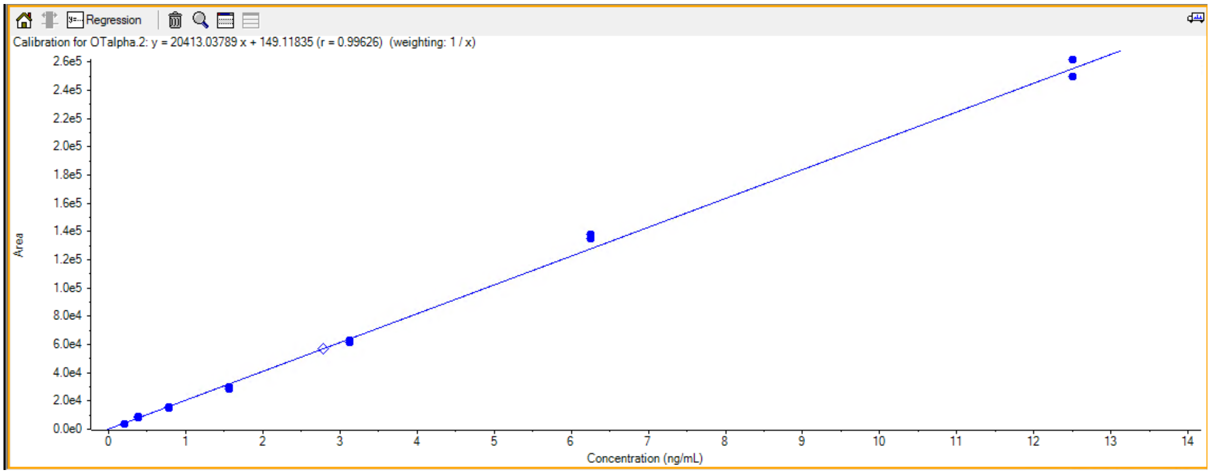

(**D**) This figure is zoomed representation of above figure C to show the points OT*α* standards between 0.195–12.5 ng/ml, along with OT*α* measured in the CPB reaction samples.

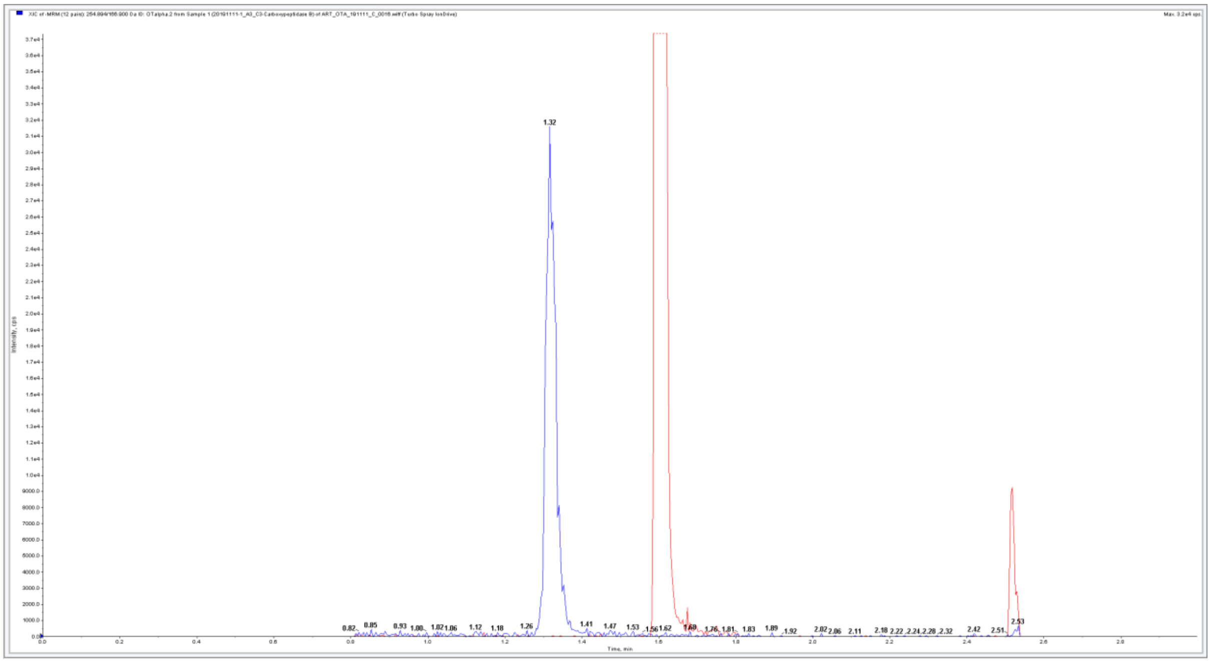

(**E**) Representation of OTα peak (blue) measured in CPB reaction sample after 180 minutes of incubation (see above Figure C and D).

Figure S19. HPLC-MS chromatograms and graphical representation of OTA (red) and OTα (blue) in the standards as well as reaction sample of carboxypeptidase B (CPB).

Table S1. First instance list of PDB identification codes of ligands and proteins identified in the virtual screening before the manual inspection.

Ligands		Proteins		
192	BB0	1AXL	2IBK	3U0D
226	BBL	1AXO	2K2G	3UAS
537	BHC	1AXU	2LGM	3VFH
636	BIK	1AXV	2NZ5	3X2S
878	BPA	1AZ1	2RH4	4AZ0
0AF	BZA	1BCR	2RHC	4CMX
0BV	BZJ	1BPS	2RHR	4DJL
0YG	BZR	1BQ4	2ROY	4E5L
18N	C16	1C85	2WF4	4FEU
1CH	CD6	1DJD	2WX0	4FUG
1HN	CXA	1DL4	2X8I	4G2Z
1HN	CZ0	1DXA	2X9H	4GIU
1T4	DBH	1ECV	2XN3	4IE7
1UZ	DNA	1EGY	2XYA	4JQA
3GQ	DR1	1ESB	2ZJW	4KKY
3J8	DSV	1F0Q	2ZMD	4L0S
46L	EL	1FYY	3BNZ	4PK6
4KL	EMO	1I7V	3BQC	4R3N
4NA	FC0	1IY7	3BY0	4R3W
5DV	HC8	1JDG	3C13	4RFR
5P3	ID8	1K9G	3CMP	4Y3S
6J1	ING	1L6M	3CSD	4YUA
7JS	JPY	1LO3	3ED0	4Z3K
7L4	KBF	1MD9	3ESS	5FDL

7ZS	KI9	1MDB	3HRR	5FJO
8P0	LQY	1MXJ	3I0A	5G2B
8SK	NPQ	1N5S	3I64	5HGP
91L	OAL	1N5T	3K3L	5IKR
9AC	OBA	1N8C	3KGM	5J1Q
9AP	PEY	1OAR	3KGN	5L4F
9NF	PKL	1PMV	3L2K	5LVL
9UP	PXC	1QBY	3NKT	5NBW
A1Z	REF	1QF0	3NKT	5OPC
A4A	RHN	1S0M	3NTY	5OX6
AA	RVC	1SDK	3PWD	5PA1
ADL	S61	1SDL	3PZH	5U6Q
ALR	TBC	1U5A	3Q9W	5V2O
AN1	TBT	1U5C	3Q9X	5V48
AN3	TI0	1UKI	3R2A	5VH0
ANF	TI2	1XC9	3R43	5WM1
AP	TMM	1Y9H	3RI3	5WM8
AZN	X8I	1YY4	3RV8	5Y2U
BAP	ZY4	1Z3F	3SAO	5YV8
		2BJM	3SH7	5ZC6
		2HMK	3SH9	6AWW
		2HML	3SQP	6O4X
		2HMM	3T1D	6RWD
		2HMN	3TF6	
		2IA6	3TZB	

Table S2. Docking scores of OTA and its similar ligands, which were identified by the ligand-based virtual screening, within the enzymes investigated in this study.

**Protein**	**Gold Score**	
	**OTA**	**Crystallographic Ligand** [a]
Carboxypeptidase A	100.1	CXA: 95.8
Carboxypeptidase T	95.7	CXA: 96.1
Carboxypeptidase B	95.3	CXA: 84.7
Serine carboxypeptidase II	54.7	FC0: 57.5
Carboxypeptidase Y	74.3	Not calculated [b]
Neprilysin	87.9	TIO: 64.5
Urokinase	64.3	9UP: 56.0
Endothiapepsin	62.4	46L: 35.4
Cathepsin A	59.6	S61: 64.7
Matrix metalloproteinase 12	59.7	DSV: 83.4
Beta-secretase 1	58.2	1HN: 99.9

a The PDB identification code of each crystallographic ligand, docked within the respective enzyme, is reported followed by the recorded docking score; b At the time of investigation the enzyme was reported in the PDB in the unbound state only
